# Relationship between Urinary *N*-Desmethyl-Acetamiprid and Typical Symptoms including Neurological Findings: A Prevalence Case-Control Study

**DOI:** 10.1371/journal.pone.0142172

**Published:** 2015-11-04

**Authors:** Jemima Tiwaa Marfo, Kazutoshi Fujioka, Yoshinori Ikenaka, Shouta M. M. Nakayama, Hazuki Mizukawa, Yoshiko Aoyama, Mayumi Ishizuka, Kumiko Taira

**Affiliations:** 1 Laboratory of Toxicology, Department of Environmental Veterinary Sciences, Faculty of Veterinary Medicine, Hokkaido University, Hokkaido, Japan; 2 Hawaii Institute of Molecular Education, Honolulu, Hawaii, United States; 3 Water Research Group, School of Environmental Sciences and Development, North-West University, Potchefstroom, South Africa; 4 Department of Environmental Veterinary Sciences, Faculty of Veterinary Medicine, Hokkaido University, Hokkaido, Japan; 5 Aoyama Allergy Clinic, Gunma, Japan; 6 Department of Anesthesiology, Tokyo Women’s Medical University Medical Center East, Tokyo, Japan; Sanford-Burnham Medical Research Institute, UNITED STATES

## Abstract

Neonicotinoid insecticides are nicotinic acetylcholine receptor agonists used worldwide. Their environmental health effects including neurotoxicity are of concern. We previously determined a metabolite of acetamiprid, *N*-desmethyl-acetamiprid in the urine of a patient, who exhibited some typical symptoms including neurological findings. We sought to investigate the association between urinary *N*-desmethyl-acetamiprid and the symptoms by a prevalence case-control study. Spot urine samples were collected from 35 symptomatic patients of unknown origin and 50 non-symptomatic volunteers (non-symptomatic group, NSG, 4–87 year-old). Patients with recent memory loss, finger tremor, and more than five of six symptoms (headache, general fatigue, palpitation/chest pain, abdominal pain, muscle pain/weakness/spasm, and cough) were in the typical symptomatic group (TSG, n = 19, 5–69 year-old); the rest were in the atypical symptomatic group (ASG, n = 16, 5–78 year-old). *N*-desmethyl-acetamiprid and six neonicotinoids in the urine were quantified by liquid chromatography-tandem mass spectrometry. The detection of *N*-desmethyl-acetamiprid was the most frequent and highest in TSG (47.4%, 6.0 ppb (frequency, maximum)), followed by in ASG (12.5%, 4.4 ppb) and in NSG (6.0%, 2.2 ppb), however acetamiprid was not detected. Thiamethoxam was detected in TSG (31.6%, 1.4 ppb), in ASG (6.3%, 1.9 ppb), but not in NSG. Nitenpyram was detected in TSG (10.5%, 1.2 ppb), in ASG (6.3%, not quantified) and in NSG (2.0%, not quantified). Clothianidin was only detected in ASG (6.3%, not quantified), and in NSG (2.0%, 1.6 ppb). Thiacloprid was detected in ASG (6.3%, 0.1 ppb). The cases in TSG with detection of *N*-desmethyl-acetamiprid and thiamethoxam were aged 5 to 62 years and 13 to 62 years, respectively. Detection of *N*-desmethyl-acetamiprid was associated with increased prevalence of the symptoms (odds ratio: 14, 95% confidence interval: 3.5–57). Urinary *N*-desmethyl-acetamiprid can be used as a biomarker for environmental exposure to acetamiprid. Further multi-centered clinical research in larger patients groups with more metabolites analysis is needed.

## Introduction

**Fig 1:**
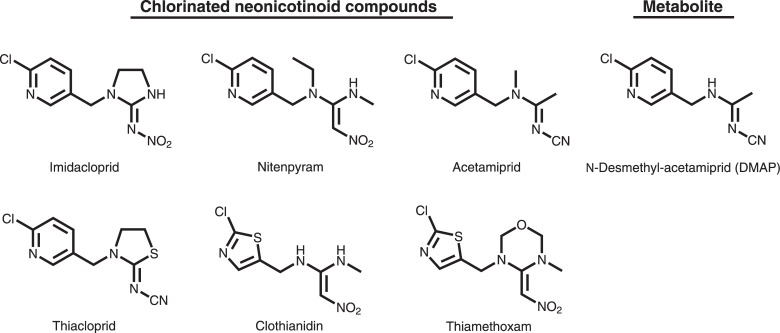
Chemical structures of six chlorinated NN insecticides and a metabolite.

Neonicotinoid (NN) insecticides are the world’s best selling insecticide class, constituting nearly 30% of the market [1], and the most widely used alternative to organophosphorus (OP), methylcarbamate, and pyrethroid insecticides in agricultural pest management, as well as domestic pest control and wood treatment [2]. NNs are systemic insecticides that are absorbed in the plant body through the root system, leaves and fruit surface, similar to phenylpyrazole insecticides [3]. NNs are believed to have a selective toxicity to insects as an agonist of α4β2 nicotinic acetylcholine receptors (nAChR) [4]. Since 1993, tons of NNs have entered the Japanese market. In 2012, 49.6 tons of acetemiprid, 36.8 tons of thiamethoxam, 66.7 tons of clothianidin, 64.9 tons of imidacloprid, 15.0 tons of thiacloprid, 7.1 tons of nitenpyram, and 152.0 tons of dinotefuran were shipped in Japan [5] (S1 Fig). The former six NNs are chlorinated compounds (Fig 1), which generally exhibit lower LD_50_ for vertebrates compared to non-chlorinated compounds, e.g. dinotefuran (S1 Table).

In Japan, chlorinated NNs are used for a wide variety of crops, and the maximum residual limit is set higher than in other countries, especially for fruits and tea leaves, with maximum being 5 ppm, 50 ppm, respectively (S2 Table). A local monitoring survey of pesticide residues in agricultural products from 1995 to 2009 fiscal year, showed residual chlorinated NNs in 101 samples of domestic conventional fruits collected at wholesale and retail markets was no more than 0.5 ppm, and the detection rate was 25.7% for acetemiprid, 11.8% for thiamethoxam and 11.8% for clothianidin [6]. Another local monitoring survey in 2012 showed residual chlorinated NNs in domestic tea leaves was no more than 0.5 ppm of clothianidin, imidacloprid, and thiamethoxam [7]. NNs are also detected in water found in nature in ppt level [8]. Environmental exposure to NNs in vertebrates including humans, as well as invertebrates, is a growing concern because of their detection frequency [9–11].

Our knowledge about adsorption, distribution, metabolism and excretion (ADME) of NNs are as follows:

Absorption: For humans NNs are absorbed through ingestion of locally grown produce, tea beverages and drinking water, dermal contact and/or inhalation by drift of pesticide spraying, treated seed sowing, other occupational procedure, in-door air pollution from structural wood treatments for termite control, and pest control for pet animals may occur [2, 3]. Major NNs and metabolites are water-soluble, have low octanol-water partition coefficients, and are highly soluble to polar solvents, such as acetone (S3 Table). They exhibit either low pKa of conjugate acids (e.g. 3.1 for nitenpyram or lower) or high pKa (e.g. 11.1 for clothianidin and imidacloprid or higher), suggesting that they exist as neutral, non-ionized species under physiological conditions. They are well absorbed by the intestinal tract actively [12], and plausibly through the airway and skin [13–18].

Distribution: They are transported via the bloodstream, pass through the blood brain barrier, blood testis barrier, and are distributed to spleen, heart, bone, lung, adrenal gland, ovary, uterus, peripheral nerve, pancreas, thyroid gland, brain, liver, kidney, skeletal muscle, adipose tissue, testis and skin [19, 20] (S4 Table). Their high affinity with globular albumin and hemoglobin are reported [21]. They are agonists of nAChR, at least of the chicken α4β2 subtype which are mainly located in the brain [4, 22]. Nicotine, imidacloprid and acetamiprid activated rat nAChRs in the neonatal cerebellar neurons at 1 μM [23]. Human nAChRs are found in the central nervous system; autonomic ganglia that dominate the gastrointestinal tract, heart, vascular smooth muscle, secretory glands, thyroid gland, adrenal gland, pupils, bronchus, liver, pancreas, kidney, bladder, genital organs, and erector pili muscles; the neuro-muscular junction on the skeletal muscle; white blood cells, airway epithelia, dermal keratinocytes, and placental cytotrophoblasts [24–26]. However, the efficacy of each NN/metabolite to each receptor is scarcely known. Some case reports of NN intoxication with nicotinic symptoms after occupational exposure or intentional intake were reported [27–29], (S5 Table). Their symptoms are mainly considered to be related to nAChR, but partially seem to be caused by co-formulated agents, such as detergents and solvents [30].

Metabolism: NNs are rapidly metabolized in the liver by phase I enzymes, e.g. cytochrome P450 (CYP) and aldehyde oxidase (AO) in mammals [31]. Phase I metabolites undergo further phase II conjugation, e.g. glutathione conjugation, glycine conjugation and glucuronidation [19, 20, 32]. NNs are also metabolized everywhere that CYP or AO are expressed e.g. red blood cells, plasma, lung, skin, brain, kidney, spleen, endocrine tissue, adipose tissue, placenta, testis, and the ovaries [33, 34]. Some NN metabolites are known to be more toxic for vertebrates than parent molecules [32] (S1 Table).

Excretion: NNs and metabolites are mostly excreted in the urine, to some extent in feces, but scarcely in the lungs [13–18]. Their low molecular weights (MW: less than 300) and water solubility suggest they pass through glomerulus freely; however, whether they are resorbed or excreted by renal proximal tubules is unknown. In a case of occupational imidacloprid intoxication renal dysfunction was reported, whereas imidacloprid was excreted in the urine for a few days [29]. Although the elimination rates of NNs in humans are unknown, clothianidin, a metabolite of thiamethoxam, exhibits two-compartment pharmacokinetics with a short distribution half-life (t_1/2_ α) of 0.88–1.89 hours and a long elimination half-life (t_1/2_ β) of 22.6–54.1 hours in Sprague Dawley rats [35].

From 2006 to 2014, we saw hundreds of patients with typical symptoms and abnormal electrocardiographic findings [36], and several thousands with atypical symptoms. Before 2006 we only observed such patients after an acetamiprid spray for pine trees in 2004 to 2005 (S1 Text). Most of the patients since 2006 recovered after the prohibition of domestic fruits and tea beverage intake in several days to weeks. The typical symptoms those patients exhibited were six subjective symptoms (headache, general fatigue, palpitations or chest pain, abdominal pain, muscle pain or muscle weakness or muscle spasm, and cough) and three objective symptoms (postural finger tremor, recent memory loss, and fever) [36] (S6 Table), which are empirically named “*neo-nicotinic symptoms*”, as well as abnormal electrocardiographic findings. Their psychiatric symptoms were pathognomonic, e.g. altered consciousness as manifested by a dreamy state; cognitive disturbances (recent memory loss with compulsive behaviors); emotional disturbance (agitation, fear or anger); psychosensory symptoms (the sudden change in the sense of smell, auditory and visual hallucinations) [36]. In those findings, recent memory loss was selected as a typical psychiatric symptom of the neo-nicotinic symptoms, which was documented and assessed by food diary before 3 days of the first visit.

In the urine of six patients with neo-nicotinic symptoms without occupational or intentional exposure to insecticides, after consecutive intake of domestic fruits and tea beverage, 6-chloro nicotinic acid (6-CNA) was detected 7.5–87.8 ppb (47.6–557 nM) [36]. 6-CNA is a common metabolite of some NNs, i.e. acetemiprid, imidacloprid, thiacloprid, nitenpyram and cycloxaprid. However, 6-CNA could not specify the original substance, and was mainly identified in the patients’ urine after more than 24 hours of the onset of the symptoms when they were close to recovery [36], (S6 Table). In addition to 6-CNA, from one of the patients within 24 hours of the onset, *N*-desmethyl-acetamiprid (DMAP) (Fig 1), an original metabolite of acetamiprid was quantified at a level of 3.2 ppb (15.4 nM) in the urine [9]. At the same time we qualitatively detected 5-hydroxy-imidacloprid (5-HIP) and *N*-desmethyl-clothianidin (DMCP) in the patients’ urine; however, we could not quantified because only DMAP is commercially available as the specific metabolite of each NN [9].

The purpose of this study was to evaluate the association between urinary DMAP and neo-nicotinic symptoms by a prevalence case-control study. The second purpose of this study was to determine six chlorinated NNs in the urine simultaneously by liquid chromatography-tandem mass spectrometry (LC/MS/MS). Four are chloropyridinyl NNs, i.e. acetamiprid, imidacloprid, nitenpyram and thiacloprid, and the rest are clothianidin and thiamethoxam; and they are chosen since we qualitatively detected some of their metabolites in the urine in the previous study.

## Materials and Methods

### Chemicals

Acetamiprid and nitenpyram were purchased from Wako Chemicals (Osaka, Japan) and imidacloprid, from Kanto Chemicals (Tokyo, Japan). Clothianidin, thiacloprid and a solution of DMAP were from Sigma-Aldrich (St. Louise, MO). Thiamethoxam was from Dr. Ehrenstorfer (Augsburg, Germany). Acetonitrile, distilled water, formic acid, sodium hydroxide, disodium hydrogen phosphate and sodium dihydrogen phosphate anhydrous were of analytical grade purchased from Kanto Chemicals (Tokyo, Japan).

### Subjects and Urine Samples

After approval by the Ethics Committee of Tokyo Women’s Medical University (No.2810), human spot urine samples were collected from patients over a period of three years who visited a clinic in Gunma prefecture, Japan, between September 2012 and March 2014, and provided written informed consent. When patients were minors or children, written informed consent was obtained from the next of the kin, caretakers, or guardians on behalf of them. The clinic is located in central Japan; two million people live in this 700 km^2^ basin with fields where rice, vegetables and fruits are grown. Inhabitants typically live in dwellings very close to agricultural fields. A physician performed systematic physical examination including neurological examination for all cases. Patients who were unawakened without any stimuli (Japanese Coma Scale II or III), whose verbal communication was limited, or who could not say their name, address, and age, were excluded.

**Fig 2:**
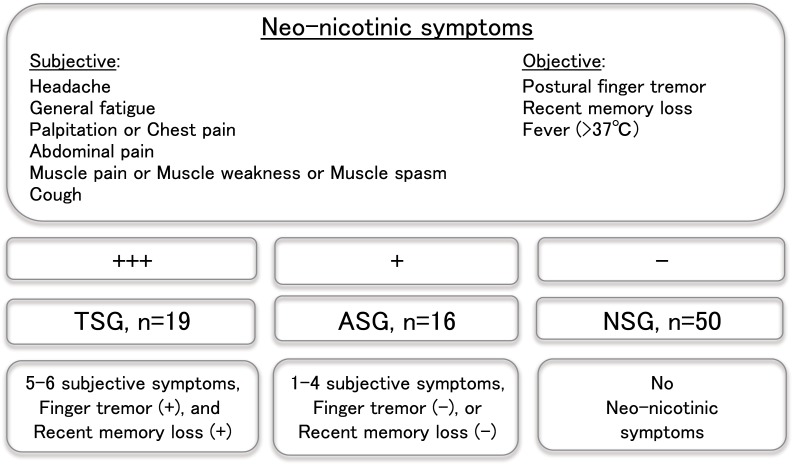
Schematic representation of inclusion criteria for each group in this study. TSG stands for the typical symptomatic group, ASG for the atypical symptomatic group and NSG for non-symptomatic group.

Thirty-five cases were enrolled from patients who showed some or all of the aforementioned nine neo-nicotinic symptoms of unknown origin. Standard 12 lead electrocardiograms were recorded for all cases. Cases were further categorized into two groups, a typical symptomatic group and an atypical symptomatic group. The typical symptomatic group (TSG, n = 19, from 5 to 69 year-old) included patients showed major objective symptoms, i.e. postural finger tremor and recent memory loss; and no less than five of six subjective symptoms, i.e. headache, general fatigue, palpitation or chest pain, abdominal pain, muscle pain or muscle weakness or muscle spasm, and cough. The atypical symptomatic group (ASG, n = 16, from 5 to 78 year-old) included patients that did not satisfy the criteria for TSG membership (Fig 2). Recent memory loss was diagnosed when the patient could not recall or fill out a questionnaire of recent meals asking what (s)he ate in the previous three days. All patients were treated supportively, and administered e.g. maltose-Ringer solution transfusion, medication such as acetaminophen for pain and fever, and lactobacillus for abdominal pain. TSG patients were advised by the treating physician not to eat locally grown fruits or drink tea beverages, and for those who made future visits to the clinic, not to consume these items until the physician confirmed that the postural finger tremor (an objective neo-nicotinic symptom) had diminished. Fifty volunteers that were sex and age-matched, without any neo-nicotinic symptoms, were recruited and referred to as the non-symptomatic group (NSG, n = 50, from 4 to 87 year-old); electrocardiograms were not recorded for this group.

Demographic data of TSG, ASG and NSG are shown in Table 1. All patients were ethnically Japanese and non-smokers.

**Table 1:** Demographic data from TSG, ASG, and NSG.

	TSG	ASG	NSG	P value^a^
**n**	19	16	50	
**Female sex (%)**	13 (68.4)	10 (62.5)	37 (74.0)	0.871
**Age (years old)**				
**≤9**	1	3	4	
**≥10, ≤14**	5	3	4	
**≥15, ≤49**	8	6	30	
**≥50, ≤64**	4	2	6	
**≥65**	1	2	6	
**mean±SD**	33.4±21.0	30.9±23.0	39.3±20.1	0.287
**min-max**	5–69	5–78	4–87	
**Subjective symptoms**				
**Number of symptoms (mean±SD)**	5.32±0.48	3.69±1.45	0	
**Headache (%)**	19 (100)	15 (93.7)	0	
**General fatigue (%)**	19 (100)	14 (87.5)	0	
**Muscle symptoms** ^b^ **(%)**	19 (100)	6 (37.5)	0	
**Chest symptoms** ^c^ **(%)**	18 (94.7)	9 (56.3)	0	
**Abdominal pain (%)**	18 (94.7)	8 (50.0)	0	
**Cough (%)**	13 (68.4)	7 (43.8)	0	
**Objective symptoms**				
**Number of symptoms (mean±SD)**	2.53±0.51	1.44±1.03	0	
**Postural finger tremor (%)**	19 (100)	13 (81.3)	0	
**Recent memory loss (%)**	19 (100)	5 (31.3)	0	
**Fever >37 centigrade (%)**	10 (52.6)	5 (31.3)	0	
**Electrocardiographic findings**				
**Number of positive patients (%)**	19 (100%)	10 (62.5%)	N/A^d^	
**Sinus tachycardia**	13	8		
**Sinus bradycardia**	3	2		
**Supraventricular arrhythmia**	0	3		
**Ventricular arrhythmia**	1	0		
**Prolongation of QT time**	2	0		
**Right bundle branch block**	6	3		
**Left bundle branch block**	1	0		

^a^ Fisher's exact probability test and Student t test for TSG vs. NSG.

^b^ Muscle symptoms include muscle pain, muscle weakness, and muscle spasm.

^c^ Chest symptoms include palpitations and chest pain.

^d^ Electrocardiography was not measured in the NSG.

Spot urine samples of approximately 50 mL were collected from 35 cases (TSG and ASG) and 50 controls (NSG) in plastic tubes and numbered for randomization. They were frozen immediately, sent to Hokkaido University, and kept in a freezer at –35°C until analysis. Recipient data were recorded and stored in a computer at Tokyo Women’s Medical University, not accessible to investigators at Hokkaido University until all analyses were complete. Blank urine samples of approximately 50 mL were collected in 2013 from healthy volunteers (n = 10) at the laboratory in plastic tubes. Blank urine samples were mixed together and kept in a freezer at –35°C until analysis. Blank urine was analyzed by LC/MS/MS in advance to make sure that no NN or interfering peak was detected, therefore the maximum levels of interfering peaks were presumably less than the limit of detection (LOD) level for each analyte.

### Preparation of LC/MS/MS Samples

Sample preparation was conducted according to a previously developed method with minor modifications to improve the number of analytes and the speed for screening a large number of samples [9]. A WAX solid-phase extraction (SPE) column (absorbent 225 mg, 60 μm, volume 2 mL, Waters, Milford, MA) was attached with a manifold and successively pre-conditioned with a column volume of acetonitrile and water. After the urine was thawed and vortex-mixed for 5 seconds, an aliquot (1 mL) of urine sample was loaded; and impurities were removed with 2 mL of 0.1 M sodium hydroxide, 2mL of 0.1 M sodium phosphate buffer (pH = 7.4) and 2 mL of water. DMAP and NNs were eluted with 2 mL of acetonitrile; and the eluate was dried under a stream of nitrogen until complete dryness. Extracts were reconstituted with 100 μL of mixture of water and acetonitrile (97/3, v/v), vortex-mixed for 10 seconds, and used for LC/MS/MS analysis immediately. Standard urine solutions were prepared with blank (non-NN-containing) urine and standard solutions of analytes. The fortified levels of concentrations for nitenpyram, thiamethoxam, acetamiprid and thiacloprid were set at 0.1, 0.3, 0.6, 0.9, and 1.2 ng/mL, for clothianidin at 0.2, 0.6, 1.2, 1.8, and 2.4 ng/mL, and for imidacloprid and DMAP at 0.5, 1.5, 3.0, 4.5, and 6.0 ng/mL. Analyte levels in the samples were determined using the external standard methods.

### Instrumentation

A Shimadzu LCMS-8040 liquid chromatograph triple quadrupole mass spectrometer (Shimadzu, Kyoto, Japan) equipped with a Luna PFP (2) column (100 Å, 2.1 x 50 mm, Phenomenex, Torrance, CA) was used for quantification. Mobile phases were water (A) and acetonitrile (B) containing 0.1% formic acid, with a linear gradient from 3% to 100% B in 8 min. The flow rate was 0.3 mL/min. The injection volume was 5 μL. The ionization mode was positive with electrospray ionization (ESI). The nebulizing gas was 3 L/min; the drying gas was 20 L/min; the desolvation line (DL) temperature was 300°C; and the heating block temperature was set to 450°C. The Multiple Reaction Monitoring (MRM) parameters for LC/MS/MS are listed in Table 2. To increase the number of analytes per one run, we used primary mass transition for each analyte for the quantitative analysis. When analytes were detected, we re-analyzed the samples using primary and secondary mass transitions for every detected analyte for qualification. The retention time for each analyte under the condition is listed in Table 3. The recovery efficient, LOD and limits of quantitation (LOQ) were determined for each analyte with six determinations of a standard urine solution [37]. LOD was calculated as:

LOD = 3 σ / S

where σ is the standard deviation of the response.

**Table 2:** Multiple Reaction Monitoring (MRM) parameters for LC/MS/MS.

Compound	Ionization mode	MRM transitions (*m*/*z*)	2nd MRM transitions (*m*/*z*)	Dwell time (ms)	Q1 pre bias (V)	Collision energy	Q3 pre bias (V)
**DMAP** ^a^	Positive	209.1/126.0	209.1/84.95	100	-14	-18	-18
**Acetamiprid**	Positive	222.7/126.0	222.7/56.1	100	-15	-21	-12
**Thiacloprid**	Positive	252.7/126.05	252.7/99.15	100	-17	-12	-19
**Imidacloprid**	Positive	256.0/209.05	256.0/239.1	100	-17	-15	-19
**Nitenpyram**	Positive	271.0/56.15	271.0/225.0	100	-20	-29	-23
**Clothianidin**	Positive	250.1/168.95	250.1/132.1	100	-17	-12	-29
**Thiamethoxam**	Positive	292.1/211.0	292.1/180.95	100	-20	-12	-19

^a^ *N*-desmethyl-acetamiprid

**Table 3:** Retention time, recovery efficiency (n = 10), limit of detection (LOD), and limit of quantification (LOQ) for DMAP and six chlorinated NNs in the urine.

Compound	Retention time (min)	Recovery (%)	LOD (ng/mL)	LOQ (ng/mL)
**DMAP** ^a^	4.06	104.9±0.5	0.59	2.0
**Acetamiprid**	4.25	95.2±5.4	0.13	0.46
**Thiacloprid**	4.48	114.5±4.7	0.02	0.05
**Imidacloprid**	4.12	93.7±3.5	0.89	3.0
**Nitenpyram**	3.47	113.7±6.4	0.19	0.64
**Clothianidin**	4.03	109.4±1.7	0.24	0.81
**Thiamethoxam**	3.66	110.5±5.2	0.13	0.39

^a^ *N*-desmethyl-acetamiprid

S is the slope of the calibration curve.

The slope S was estimated from the calibration curve of each analyte. The estimate of σ was carried out using samples containing an analyte. LOQ was calculated as:

LOQ = 10 σ / S

The concentrations of analytes in the recovery test were set at 0.6 ng/mL for nitenpyram, thiamethoxam, acetamiprid and thiacloprid, at 1.2 ng/mL for clothianidin, and at 3.0 ng/mL for imidacloprid and DMAP.

### Urine Creatinine and Cystatin C Analysis

Urine creatinine (u-Cr) and cystatin C (u-CysC) in the urine samples of TSG, ASG, and NSG were analyzed by a contract research organization (Ikagaku Co., LTD, Kyoto, Japan), except for three of TSG and one of ASG because the sample volume was too small. u-CysC was analyzed by ELISA method, and u-Cr by enzyme method used CICALIQUID-S CRE (KANTO CHEMICAL). u-CysC/u-Cr ratio (UCCR, the unit is μg/mmol Cr) is a good clinical indicator of tubular dysfunction [38] and the reference values is less than 8 μg/mmol Cr. In normal renal tubules, creatinine is not resorbed but cystatin C is more than 99%. When renal tubular reabsorption reduces by tubular dysfunction, u-CysC reabsorption also decreases, u-CysC concentration increases, and causes UCCR elevation.

### Data Analysis

XLSTAT version 2014.4.06 (Addinsoft, New York, NY) was used for statistical analysis. Fisher's exact probability test or the Mann-Whitney test for non-parametric data and the Student’s two-group t test for parametric data were used to compare groups. The Kruskal-Wallis test was used for multiple comparison of each symptom’s prevalence between the two groups. The significance level was set at *P* = 0.05.

## Results

DMAP and six chlorinated NNs were simultaneously quantified in human urine by LC/MS/MS with a satisfactory linearity within the range of standard solutions and quantitative recovery efficiency for each analyte. The typical chromatograms for DMAP and six chlorinated NNs obtained from a standard urine solution are shown in S2 Fig. LOQ for the analytes ranged from 0.05 ng/mL (thiacloprid) to 3.0 ng/mL (imidacloprid) (Table 3). DMAP was more frequently detected than other analytes, followed by thiamethoxam, nitenpyram, thiacloprid and clothianidin in the urine samples (Table 4). Representative chromatograms of the urine extract in a patient with simultaneous detection of DMAP and thiamethoxam are shown in S3 Fig. Representative chromatograms of the urine extract in a patient with detection of thiamethoxam for quantification and qualification are shown in S4 Fig.

**Table 4:** Number of individual DMAP and NNs detected in urine.

Compound	Number analyzed	Number detected	Number quantified	Range (ng/mL)	Median (ng/mL)	Quartile (ng/mL)
**DMAP** ^a^	85	14	5	0.62–6.0	1.2	0.77
**Acetamiprid**	85	0	0	-	-	-
**Thiacloprid**	85	1	1	0.14	0.14	0
**Imidacloprid**	85	0	0	-	-	-
**Nitenpyram**	85	4	1	0.25–1.2	0.52	0.18
**Clothianidin**	85	1	1	1.6	1.6	1.6
**Thiamethoxam**	85	7	5	0.26–1.9	0.98	0.43

^a^ *N*-desmethyl-acetamiprid

**Fig 3:**
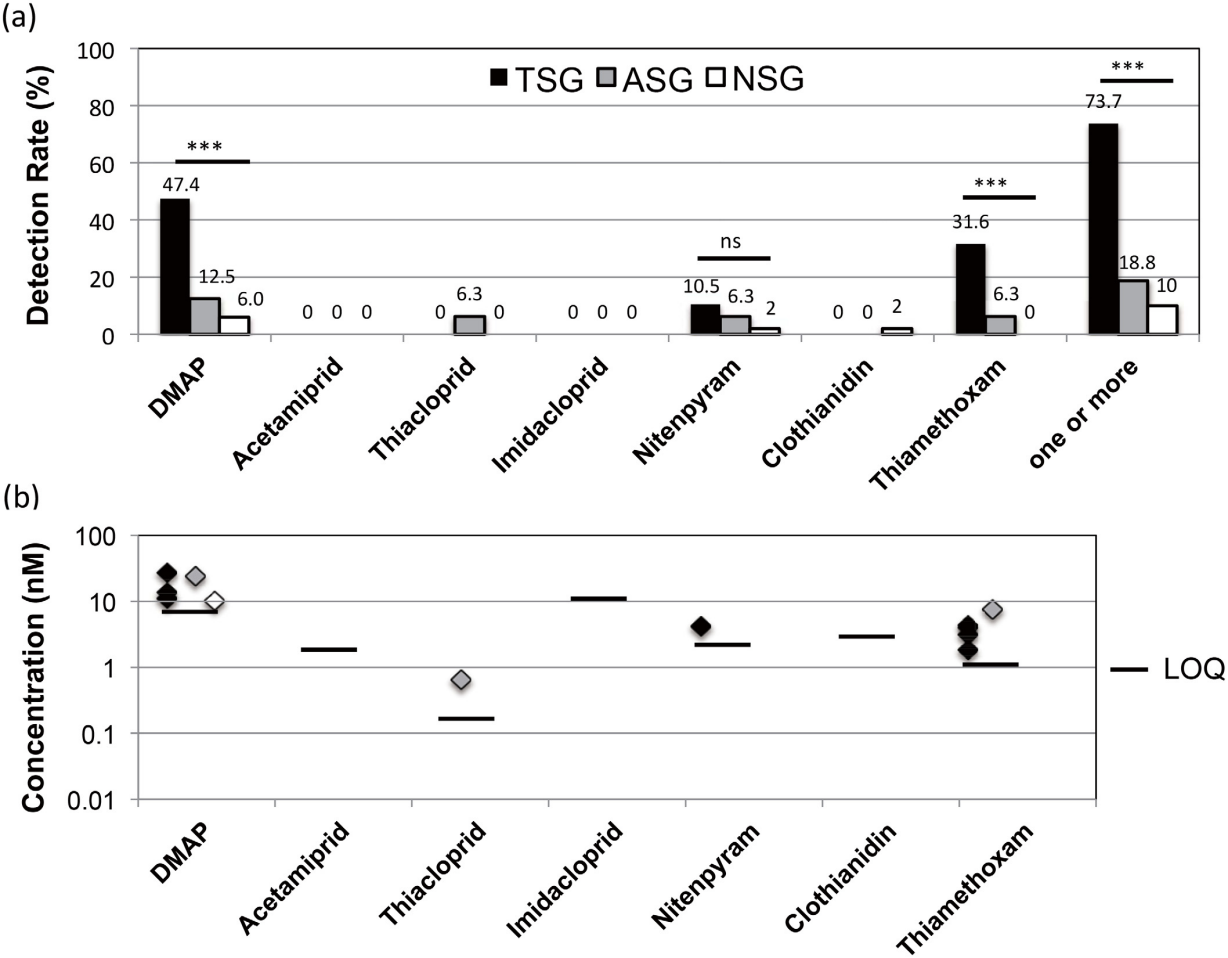
Detection rates and quantified levels of DMAP and NNs in TSG, ASG and NSG. The upper part (a) is the detection rate, and the lower part (b) is the quantified levels. TSG stands for the typical symptomatic group, ASG for the atypical symptomatic group and NSG for the non-symptomatic group. “***” stands for p < 0.0001, and NS stands for not significant.

The detection of DMAP, thiamethoxam and nitenpyram were the most frequent in TSG, followed by ASG, and NSG (Fig 3). Thiacloprid was detected only in ASG, and clothianidin was detected in NSG (Table 5). The number of simultaneous detection of multiple NNs was three out of 19 (15.8%) in TSG (DMAP and thiamethoxam in two cases; thiamethoxam and nitenpyram in a case) and two out of 16 (12.5%) in ASG (DMAP, thiamethoxam, and clothianidin in a case; thiacloprid and nitenpyram in a case). The number of detection for DMAP in the urine was 9 (47.4%) in TSG, 2 (12.5%) in ASG, and 3 (6.0%) in NSG. The prevalence odds ratio of the neo-nicotinic symptoms of TSG for the detection of DMAP concentrations in urine was 14 (95% confidence interval = 3.5–57) and that of ASG was 2.4 (95% confidence interval = 0.40–13).

**Table 5:** Number of individual DMAP and NNs detected in the urine of TSG, ASG, and NSG.

	TSG	ASG	NSG	p value ^a^	Odds Ratio ^a^ [95%CI]
**n**	19	16	50		
**>LOD** ^b^ **(n (%))**					
**DMAP** ^c^	9 (47.4)	2 (12.5)	3 (6.0)	<0.0001	14 [3.5–57]
**Acetamiprid**	0 (0.0)	0 (0.0)	0 (0.0)		
**Thiacloprid**	0 (0.0)	1 (6.3)	0 (0.0)		
**Imidacloprid**	0 (0.0)	0 (0.0)	0 (0.0)		
**Nitenpyram**	2 (10.5)	1 (6.3)	1 (2.0)	0.148	5.7 [0.71–47]
**Clothianidin**	0 (0.0)	1 (6.3)	1 (2.0)		
**Thiamethoxam**	6 (31.6)	1 (6.3)	0 (0.0)	<0.0001	Not calculated
**One or more**	14 (73.7)	3 (18.8)	5 (10.0)	<0.0001	25 [6.7–95]
**>LOQ** ^d^ **(n (%))**					
**DMAP**	3 (15.8)	1 (6.3)	1 (2.0)		
**Thiacloprid**	0 (0.0)	1 (6.3)	0 (0.0)		
**Nitenpyram**	1 (5.3)	0 (0.0)	0 (0.0)		
**Clothianidin**	0 (0.0)	0 (0.0)	0 (0.0)		
**Thiamethoxam**	4 (21.1)	1 (6.3)	0 (0.0)		
**Maximum concentration (ng/mL)**					
**DMAP**	6.0	4.4	2.2		
**Thiacloprid**	<LOD	0.14	<LOD		
**Nitenpyram**	1.2	<LOD	<LOD		
**Clothianidin**	<LOD	<LOQ	1.6		
**Thiamethoxam**	1.4	1.9	<LOD		

^a^TSG vs, NSG,

^b^LOD: Lowest level of detection,

^c^ *N*-desmethyl-acetamiprid

^d^Lowest level of quantification,

u-Cr and u-CysC were measured in sixteen of 19 TSG, in fifteen of 16 ASG and in all NSG, whereas DMAP was detected in 1 TSG and 1 ASG out of four unmeasured samples. The result of u-Cr, u-CysC and UCCR is shown in S7 Table. Creatinine-corrected DMAP concentration was higher in 3 TSG cases (2.8, 3.0, and 3.6 nmol/mmol Cr) than in a NSG patient (1.3 nmol/mmol Cr). Creatinine-corrected thiamethoxam concentrations in 3 TSG and 1 ASG cases were 0.24, 0.44, 0.58, and 0.35 nmol/mmol Cr, respectively (S8 Table). The concentration level of u-Cr and u-CysC was not significantly different between TSG and NSG and the number of UCCR more than the reference value was 2 in TSG (12.5%), 3 in ASG (20.0%), and 4 in NSG (8.0%). However, UCCR was significantly higher in TSG compared to NSG (p = 0.033, Mann-Whitney test). The detection rate of DMAP was higher in the high UCCR group in TSG and NSG, but the trend was not observed in that of thiamethoxam in TSG (S7 Table).

An age and gender analysis of cases with DMAP and NNs detection is shown in Table 6. DMAP and thiamethoxam were detected in all three age groups, i.e. under 15, 15–49, over 50 years old, of TSG.

**Table 6:** Age and gender analysis of cases with DMAP and NNs detection by young (≤14), intermediate-age (≥15, ≤49) and aged (≥50) subgroups.

	TSG		ASG		NSG	
	n (M, F)	%	n (M, F)	%	n (M, F)	%
**≤14 year-old**	6 (2, 4)	100.0	6 (3, 3)	100.0	8 (7, 1)	100.0
**DMAP** ^a^	3 (1, 2)	50.0	0	0	0	0
**Thiacloprid**	0	0	0	0	0	0
**Nitenpyram**	0	0	0	0	0	0
**Clothianidin**	0	0	0	0	0	0
**Thiamethoxam**	1 (0, 1)	16.7	0	0	0	0
**≥15, ≤49 year-old**	8 (5, 3)	100.0	6 (1, 5)	100.0	30 (4, 26)	100
**DMAP**	3 (1, 2)	37.5	2 (0, 2)	33.3	3 (1, 2)	10.0
**Thiacloprid**	0	0	1 (0, 1)	16.7	0	
**Nitenpyram**	1 (1, 0)	12.5	1 (0, 1)	16.7	0	0
**Clothianidin**	0	0	1 (0, 1)	16.7	1 (0, 1)	5.0
**Thiamethoxam**	4 (2, 2)	50.0	1 (0, 1)	16.7	0	0
**≥50 year-old**	5 (0, 5)	100.0	3 (1, 2)	100.0	12 (2, 10)	100
**DMAP**	3 (0, 3)	60.0	0	0	0	0
**Thiacloprid**	0	0	0	0	0	0
**Nitenpyram**	1 (0, 1)	20.0	0	0	1 (1, 0)	8.3
**Clothianidin**	0	0	0	0	0	0
**Thiamethoxam**	1 (0, 1)	20.0	0	0	0	0

^a^ *N*-desmethyl-acetamiprid

All TSG members and 62.5% of the ASG members had more than one electrocardiographic findings, including sinus tachycardia, sinus bradycardia, supraventricular arrhythmia, ventricular arrhythmia, and right bundle branch block, or QT prolongation (Table 1). The most prevalent electrocardiographic finding for TSG or ASG cases with detection of DMAP in the urine (n = 11) was sinus tachycardia (9, 81.8%) (S8 Table). The most prevalent symptoms in the cases of TSG or ASG with detection of DMAP in the urine (n = 11) was postural finger tremor, headache, general fatigue and muscle pain/muscle weakness/muscle spasm (11, 100%), followed by palpitations/chest pain, abdominal pain (10, 90.9%), recent memory loss (9, 81.8%), fever (6, 54.5%), and cough (5, 45.6%) (Table 1). Representative questionnaires on recent meals are shown in S5 Fig.

The summary of clinical course in TSG or ASG patients is shown in Table 7. Details are shown in S8 Table. No case was a member of the farmer’s family. In fifteen cases with detection of DMAP or thiamethoxam, four cases did not visit the clinic again. Nine of those cases had improvement of all subjective symptoms, postural finger tremor and recent memory loss in 1–180 days after prohibiting consumption of locally grown fruits and tea beverages, as well as electrocardiographic findings. However, a 16-year-old boy (Case 5 in TSG, S8 Table) with a simultaneous detection of DMAP and thiamethoxam in the urine and a 62-year-old woman (Case 9 in TSG, S8 Table) did not experience the improvement of postural finger tremor or other neo-nicotinic symptoms in spite of the dietary prohibitions.

**Table 7:** The summary of clinical course in TSG and ASG patients.

	TSG	ASG
n (total, (number of DMAP or thiamethoxam detected))	19 (13)	16 (2)
**Onset of neo-nicotinic symptoms (days ago)** ^a^		
**≤7**	6 (4)	6 (1)
**>7, ≤30**	2 (2)	4 (1)
**>30, ≤90**	2 (2)	1 (0)
**>90**	7 (4)	1 (0)
**Unknown**	2 (1)	4 (0)
**median±quartile**	45±182	14±8
**Number of days for improvement (days)**		
**≤7**	4 (4)	0 (0)
**>7, ≤30**	2 (2)	3 (0)
**>30, ≤90**	2 (1)	3 (0)
**>90**	3 (2)	1 (0)
**Not improved**	4 (2)	0 (0)
**Unknown**	4 (2)	9 (2)
**median±quartile**	52±95	32±10
**Intake of fruits (>500g/day) or tea beverage (>500mL/day)** ^a^		
**Tea beverage**	10 (7)	10 (1)
**Juice (fruits or vegetable)**	4 (3)	1 (0)
**Fruits**	3 (3)	9 (1)
**Unknown**	3 (3)	1 (0)
**Comorbidity**		
**Low conscious level, JCS-1**	2 (1)	0 (0)
**Electromagnetic sensitivity**	1 (1)	0 (0)
**Auditory and visual hallucination**	1 (1)	0 (0)
**Anxiety**	1 (1)	0 (0)
**Sleepless**	1 (1)	0 (0)
**Orthostatic dysregulation**	1 (1)	0 (0)
**Fibromyalgia**	1 (1)	0 (0)
**Diabetes mellitus**	1 (1)	0 (0)
**Hyperlipidemia**	1 (1)	0 (0)
**Edema**	1 (1)	1 (0)
**Nausea**	1 (1)	0 (0)

^a^ Reported by patients or family.

## Discussion

In this study we discovered that 1. DMAP and thiamethoxam were frequently detected in the urine of the patients with neo-nicotinic symptoms, 2. The prevalence odds ratio of the neo-nicotinic symptoms for the detection of urinary DMAP in TSG was significantly higher than NSG, 3. UCCR was significantly higher in TSG than in NSG. The results suggest that the occurrence of neo-nicotinic symptoms in the general population is related to environmental exposure to NNs, at least acetamiprid and thiamethoxam. Urinary DMAP can be a specific biomarker of acetamiprid exposure; and consumption of local produce is a plausible source of exposure unless there is an incidental exposure, such as in aerial spraying or domestic treatment.

The frequent detection of urinary DMAP without acetamiprid implies that environmentally exposed acetamiprid is readily metabolized in humans. Thiamethoxam seems to be poorly metabolized in humans at low levels of environmental exposure. In the literature, thiamethoxam is metabolized by the human liver CYPs and not metabolized by human liver AOs [31]. DMAP and thiamethoxam can be good biomarkers of environmental NNs exposure related to neo-nicotinic symptoms. DMAP and six chlorinated NNs were not detected in 28% of TSG; however, their exposure to NNs cannot be excluded because of the lack of proper biomarkers for each NN, which is a limitation of this study. The metabolism of clothianidin and imidacloprid in humans may be relatively fast to yield subsequent metabolites, e.g. DMCP from clothianidin and 5-HIP from imidacloprid [2, 9, 19, 20]. The development of analytical methods for NN metabolites other than DMAP needs to be pursued.

A high prevalence of urinary DMAP and thiamethoxam detection in the patients with neo-nicotinic symptoms at ppb level suggests two major possibilities. One is that something other than NN interfered with the renal tubular reabsorption of DMAP and thiamethoxam, and caused an increase in DMAP and thiamethoxam excretion in urine and neo-nicotinic symptoms, while the environmental acetemiprid and thiamethoxam exposure was in the normal ranges. However, in the same level of UCCR, detection ratio of DMAP was far higher in TSG than in NSG. We, therefore, conclude that renal tubular dysfunction is not the main reason for why DMAP and thiamethoxam were detected in the urine.

Glyphosate has recently attracted attention, as one of the toxic environmental chemicals, because of the increased use of those toxicants is correlated with many health problems including chronic kidney disease [39]. The shipment of pesticides in Gunma and Japan are almost constant in glyphosate, imidacloprid, and acetemiprid since the middle of 1990’s, but has been increasing in thiamethoxam, clothianidin, and dinotefuran since 2001 here after neo-nicotinic symptoms emerged (S1 Fig). It does not seem feasible that glyphosate, a herbicide with cytotoxicity [40], can directly cause neo-nicotinic symptoms with other neuropsychiatry symptoms and electrocardiographic abnormality, which almost disappeared with the prohibition of specific kinds of food.

Another possibility is that significant and/or prolonged exposure to acetemiprid or thiamethoxam caused neo-nicotinic symptoms with abnormal electrocardiogram, an increased excretion of DMAP or thiamethoxam and a reduced renal tubular reabsorption. We can find some evidence to support this hypothesis in this study and in the literature.

### Accumulation of NNs in Human Body

The creatinine corrected concentration of DMAP was higher in TSG than in NSG, and although a one-compartment model was proposed by Bedneska on toxicokinetics of thiamethoxam [41], a two-compartment model was used and is commonly applied to nAChR agonists in humans and experimental animals, e.g. clothianidin and pozanicline (ABT-089) [42], as well as other receptor agonists, such as dopamine [43]. Continuously exposed NNs might be distributed in a non-uniform manner and accumulate in slow compartments, resulting in high concentrations around nAChR. The concentrations of DMAP in the brain, in the patients with neo-nicotinic symptoms, could be higher than that in the urine, as observed in ABT-089. Peak concentrations for ABT-089 are approximately 10-fold higher in the brain than in the plasma by oral administration [42]. The two-compartment model is consistent with the fact that it took several days to months for the majority of TSG before the onset of symptoms and for the recovery of their neo-nicotinic symptoms (Table 7), in contrast to that of the acute case of acetamiprid intoxication, which was within 24 hours [28].

nAChR and polar tissues like proteins or phospholipid bilayer are candidates of slow compartments. NNs have a higher solubility in polar solvents, such as acetone as shown in S3 Table. For example, the solubility of thiamethoxam in acetone, water and octanol is 48, 4.1, and 0.62 g/L, respectively. High affinity of NN with globular albumin and hemoglobin are reported [21]. NNs appear to bind to multiple sites on membranes of neural tissues in variant insect species [3]. It is noteworthy that the exposure time amplifies the toxicity of NN in bees, i.e. micro-colonies of bumblebees fed with imidacloprid showed a phenomenon [44]: at one tenth of the concentration of the toxin in feed, it took twice as long to produce 100% mortality in a bumblebee micro colony, and at a 100 times lower dose, it took about four times longer to produce 100% mortality.

Regarding the suspected food with a high MRL in Japan (S2 Table), tea beverages were most frequently consumed in 8 TSG cases, followed by vegetable or fruit juice 3 cases, and fruits 3 cases, respectively. Estimation of the exposed dose of acetemiprid from every dose of food and beverages for more than a few months is impossible to do in clinical settings; however, some monitoring survey in Japan suggest remarkably high level exposure of NNs is not likely but continuous exposure may occur [6–8].

### Neo-Nicotinic Symptoms with Electrocardiographic Abnormality and NNs

We speculate that the direct agonistic action on nAChR or modulation of the immune systems [45] could be the mode of action of NNs for neo-nicotinic symptoms; however, our study provided no evidence of this. In the literature, neo-nicotinic symptoms, other psychiatric symptoms and their electrocardiographic changes (sinus tachycardia, sinus bradycardia, supraventricular arrhythmia, ventricular arrhythmia, QT prolongation, and ST-T change) partially resemble rare cases of autoimmune disease, i.e. myasthenia gravis (MG) or autoimmune autonomic ganglionopathy (AAG) with neuropsychiatry symptoms or myocarditis, caused by nM level of anti-nAChR antibody [46–48]. In a local area of Japan, an increased incidence of MG onset in the elderly with low anti-nAChR antibody titers, less autoimmune overlaps, and a nearly non complete stable remission with or without thymectomy was reported in 2009 [47].

Gender seems to be one of the factors that neo-nicotinic symptoms manifest. In this study, more than 60% were women in TSG and ASG. This trend is consistent with our previous study [9, 36]. Age dependency was not clear in our studies. However the involvement of young patients suggests the adverse effect of NNs on the developing brain; and that of aged patients suggests the effect of NNs on the brain with cognitive function problems. Inter-individual differences in genetic and epigenetic characteristics can be considerable like slow metabolizers of nicotine with a CYP2A6 genotype [49]. Little is known whether highly toxic NN metabolites develop in the human body under some conditions [32]. The effect of coformulated substances for each NN is also recently of concern [30].

### Renal Dysfunction, especially Reductions of Renal Tubular Reabsorption by NNs

The fact that UCCR was significantly higher in TSG than in NSG means NNs might be a risk of renal tubular dysfunction. NNs may accumulate in kidney after consecutive intake of contaminated food, because residual NNs are detected in the kidneys 24–96 hours after a single administration of NNs in animal studies (S4 Table). Nicotine administration induced increases in proteinuria and glomerular injury score in rats with 5/6 nephrectomies, which were prevented by co-administration of an α7-nAChR selective antagonist, methyllicaconitine [50]. Further investigation and follow-up of patients with neo-nicotinic symptoms is needed, especially using more precise urinary and blood biomarkers, to test the hypothesis of NN-induced renal dysfunction.

## Conclusion

The association between urinary *N*-desmethyl-acetemiprid, as well as chlorinated NNs and clinical symptoms in 85 subjects from the Japanese general population was evaluated by a prevalence case-control study. The detection of *N*-desmethyl-acetemiprid was correlated with the simultaneous exhibition of postural finger tremor, recent memory loss, headache, general fatigue, chest symptoms, abdominal pain, and muscle symptoms with electrocardiographic findings. Their symptoms mostly continued for several days to months after prohibiting the consumption of locally grown produce. The results of this study suggest further study on urinary NN metabolites including DMAP and others in humans.

## Supporting Information

S1 Fig Annual shipment of neonicotinoid insecticides and glyphosate in Japan and in Gunma prefecture. (PDF)

S2 Fig Representative LC/MS/MS of DMAP and six chlorinated NNs obtained from a standard urine solution. (PDF)

S3 Fig Representative LC/MS/MS of the urine extract in a patient with simultaneous detection of DMAP (pink) and thiamethoxam (black). (PDF)

S4 Fig Representative LC/MS/MS of the urine extract in a patient with quantitative (black) and qualitative (pink) detection of thiamethoxam. (PDF)

S5 Fig Representative questionnaires on recent meals. (PDF)

S1 Table The names, common names, alternative abbreviations in literature and oral toxicity of seven neonicotinoid insecticides and metabolites. (PDF)

S2 Table Maximum residue levels (MRLs) of neonicotinoid insecticides in Japan, in compared to EU, US, and CODEX. (PDF)

S3 Table Physicochemical characteristics of neonicotinoid insecticides. (PDF)

S4 Table Residual Radioactivity of neonicotinoids in Tissues and Organs of Male and Female Rats after a Single Oral Administration of radiolabeled each neonicotinoid. (PDF)

S5 Table Case reports analysis of acute imidacloprid and acetamiprid intoxication, and those toxic doses. (PDF)

S6 Table Time course of 6-chrolonicotinic acid (6-CNA) and the maximum 6-CNA concentrations in the urine sample of eleven patients, and their subjective symptoms, clinical findings, electrocardiogram findings, and food/beverage intake. (PDF)

S7 Table Urinary creatinine, urinary cystatin C and urinary creatinine / cystatin C ratio (UCCR) in TSG, ASG and NSG. (PDF)

S8 Table The clinical course of TSG and ASG and urinary levels of DMAP and NNs. (PDF)

S1 Text Human neonicotinoids exposure in Japan. (PDF)
