# Supplementary material for: Relationship between Urinary N-Desmethyl-Acetamiprid and Typical Symptoms including Neurological Findings: A Prevalence Case-Control Study
Source: PLoS One. 2015 Nov 4;10(11):e0142172. doi: 10.1371/journal.pone.0142172 (PMC4633099; doi:10.1371/journal.pone.0142172)
Supplement: S1 Fig — (PDF) [file pone.0142172.s001.pdf]

Supporting Information

Relationship between urinary *N*-desmethyl-acetamiprid and typical symptoms including neurological findings: A prevalence case-control study

Jemima Tiwaa Marfo[1], Kazutoshi Fujioka[2], Yoshinori Ikenaka[1, 3], Shouta M. M. Nakayama[1], Hazuki Mizukawa[4], Yoshiko Aoyama[5], Mayumi Ishizuka[1], Kumiko Taira[6*]

[1] Laboratory of Toxicology, Department of Environmental Science, Faculty of Veterinary Medicine, Hokkaido University, Hokkaido, Japan

[2]Hawaii Institute of Molecular Education, Hawaii, US

[3]Water Research Group, School of Environmental Sciences and Development, North-West University, South Africa

[4]Department of Environmental Science, Faculty of Veterinary Medicine, Hokkaido University, Hokkaido, Japan

[5]Aoyama Allergy Clinic, Gunma, Japan

[6]Department of Anesthesiology, Tokyo Women's Medical University Medical Center East, Tokyo, Japan

S1 Fig. Annual shipment of neonicotinoid insecticides and glyphosate in Japan and in Gunma prefecture.

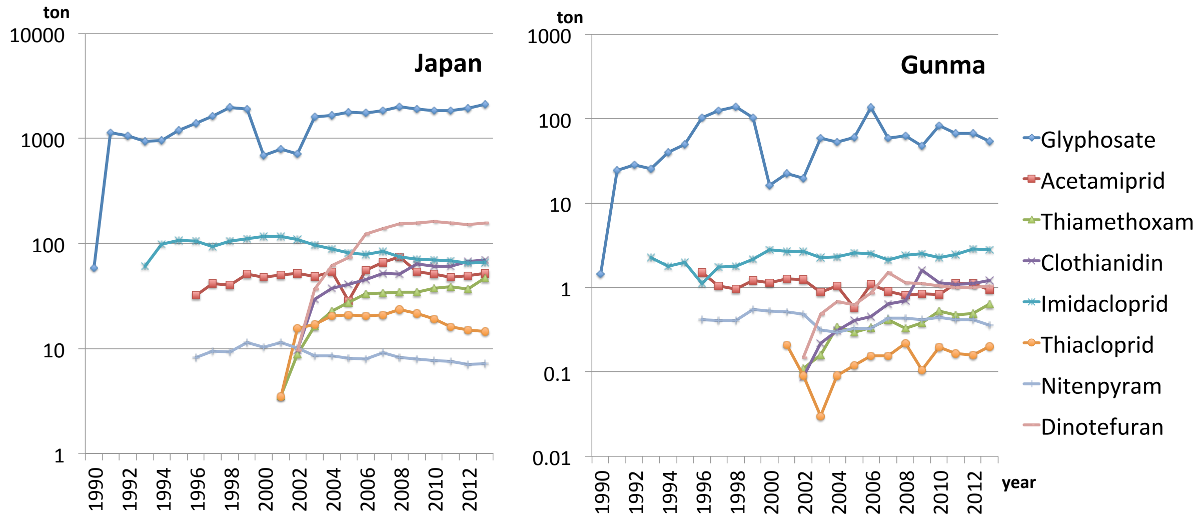

Reference: Plant Products Safety Division & Plant Protection Division, Food Safety and Consumer Affairs Bureau, Ministry of Agriculture, Forestry and Fisheries. Noyaku Yoran 1991; 1992; 1993; 1994; 1995; 1996; 1997; 1998; 1999; 2000; 2001; 2002; 2003; 2004; 2005; 2006; 2007; 2008; 2009; 2010; 2011; 2012; 2013. Tokyo: Japan Plant Protection Association. 1992-2014.
