# Supplementary material for: Relationship between Urinary N-Desmethyl-Acetamiprid and Typical Symptoms including Neurological Findings: A Prevalence Case-Control Study
Source: PLoS One. 2015 Nov 4;10(11):e0142172. doi: 10.1371/journal.pone.0142172 (PMC4633099; doi:10.1371/journal.pone.0142172)
Supplement: S5 Fig — (PDF) [file pone.0142172.s005.pdf]

Supporting Information

Relationship between urinary *N*-desmethyl-acetamiprid and typical symptoms including neurological findings: A prevalence case-control study

Jemima Tiwaa Marfo[1], Kazutoshi Fujioka[2], Yoshinori Ikenaka[1, 3], Shouta M. M. Nakayama[1], Hazuki Mizukawa[4], Yoshiko Aoyama[5], Mayumi Ishizuka[1], Kumiko Taira[6*]

[1] Laboratory of Toxicology, Department of Environmental Science, Faculty of Veterinary Medicine, Hokkaido University, Hokkaido, Japan

[2]Hawaii Institute of Molecular Education, Hawaii, US

[3]Water Research Group, School of Environmental Sciences and Development, North-West University, South Africa

[4]Department of Environmental Science, Faculty of Veterinary Medicine, Hokkaido University, Hokkaido, Japan

[5]Aoyama Allergy Clinic, Gunma, Japan

[6]Department of Anesthesiology, Tokyo Women's Medical University Medical Center East, Tokyo, Japan

S5 Fig. Representative questionnaires on recent meals.

Recent memory loss (+): The patients could not fill out contents of meals in the previous three days.

Case A: 11 years old female could not recall what she ate for lunch two days ago or before; and DMAP was quantified at 3.6 nmol/mmol Cr in her urine.

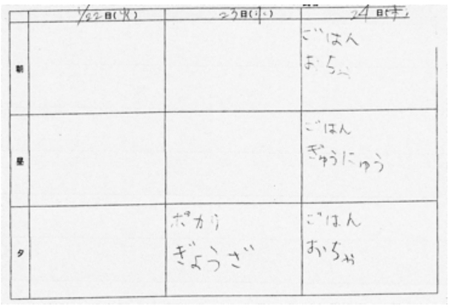

	1/22日(水)	23日(木)	24日(金)
朝			ごはん おちゃ
昼			ごはん ぎゅうにゅう
夕		ポカリ ぎょうざ	ごはん おちゃ

Meal	3 days before	2 days before	1 day before
The first			Rice, tea
The second			Rice, milk
The third		Isotonic water, meat dumpling	Rice, tea

Case B: a 62-year-old female could not recall what she ate for dinner two or three days ago or lunch three days ago; and thiamethoxam (0.24 nmol/mmol Cr) and nitenpyram (0.54 nmol/mmol Cr) were quantified in her urine.

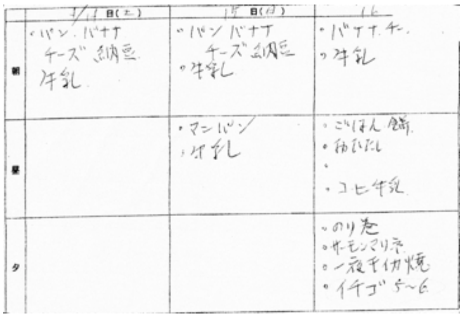

	[illegible]	15日	16
朝	・パン、バナナ チーズ 納豆 牛乳	・パン バナナ チーズ 納豆 ・牛乳	・バナナ チーズ ・牛乳
昼		・アンパン ・牛乳	・ごはん 鍋 ・おひたし ・コーヒー牛乳
夕			・のり巻 ・サーモンマリネ ・一夜干イカ焼 ・イチゴ 5~6

Meal	3 days before	2 days before	1 day before
The first	Bread, banana, cheese, natto, milk	Bread, banana, cheese, natto, milk	Banana, cheese, milk
The second		Bean-jam bun, milk	Rice, pot cooking, boiled vegetable, milk coffee
The third			Sushi, malinard salmon, grilled squid, strawberry

---

Recent memory loss (−): The patients could fill out contents of meals in the previous three days.

Case C: an 11-year-old female.

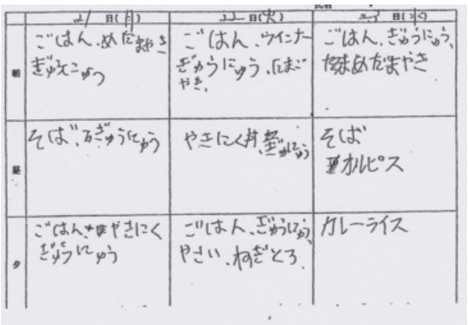

	[illegible]	[illegible]	[illegible]
朝	ごはん、めだまやき ぎゅうにゅう	ごはん、ウインナー ぎゅうにゅう たまごやき	ごはん、ぎゅうにゅう、めだまやき
昼	そば、ぎゅうにゅう	やきにく丼 ぎゅうにゅう	そば カルピス
夕	ごはん やきにく ぎゅうにゅう	ごはん、ぎゅうにゅう やさい、ねぎとろ	カレーライス

Meal	3 days before	2 days before	1 day before
The first	Rice, grilled egg, milk	Rice, sausage, milk, grilled egg	Rice, milk, grilled egg
The second	Buckwheat noodle, milk	Grilled beef rice, milk	Buckwheat noodle, yogurt drink
The third	Rice, grilled beef, milk	Rice, milk, vegetables, tuna &Welsh onion	Curry and rice

Case D: a 50-year-old male skipped breakfast two days ago and remembered it.

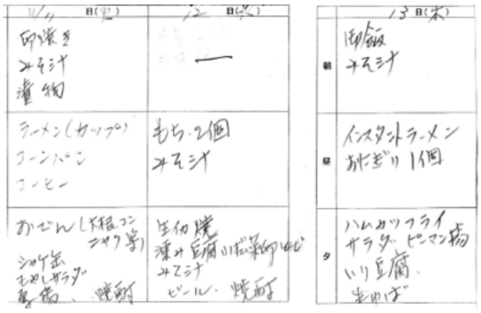

	11日	12日	13日(木)
朝	卵焼き みそ汁 漬物	—	御飯 みそ汁
昼	ラーメン(カップ) コーンパン コーヒー	もち 2個 みそ汁	インスタントラーメン おにぎり 1個
夕	おでん(大根 コンニャク) シャケ缶 もやしサラダ 厚揚 焼酎	生イカ焼 [illegible] みそ汁 ビール 焼酎	ハムカツフライ サラダ ピーマン炒 いり豆腐 ゆば

Meal	3 days before	2 days before	1 day before
The first	Grilled egg, miso soup, pickles	Skipped	Rice, miso soup
The second	Chinese noodle, corn bread, coffee	2 piece of rice cake, miso soup	Chinese noodle, a rice ball
The third	Oden (radish, konnyaku), canned salmon, beans sprout salad, fried tofu, spirit	Grilled squid, dried tofu, komatsuna, miso soup, bier, spirit	Fried ham, salad, fried pepper, grilled tofu, yuba
