# Supplementary material for: Relationship between Urinary N-Desmethyl-Acetamiprid and Typical Symptoms including Neurological Findings: A Prevalence Case-Control Study
Source: PLoS One. 2015 Nov 4;10(11):e0142172. doi: 10.1371/journal.pone.0142172 (PMC4633099; doi:10.1371/journal.pone.0142172)
Supplement: S1 Table — (PDF) [file pone.0142172.s006.pdf]

Supporting Information

Relationship between urinary *N*-desmethyl-acetamiprid and typical symptoms including neurological findings: A prevalence case-control study

Jemima Tiwaa Marfo[1], Kazutoshi Fujioka[2], Yoshinori Ikenaka[1, 3], Shouta M. M. Nakayama[1], Hazuki Mizukawa[4], Yoshiko Aoyama[5], Mayumi Ishizuka[1], Kumiko Taira[6*]

[1] Laboratory of Toxicology, Department of Environmental Science, Faculty of Veterinary Medicine, Hokkaido University, Hokkaido, Japan

[2]Hawaii Institute of Molecular Education, Hawaii, US

[3]Water Research Group, School of Environmental Sciences and Development, North-West University, South Africa

[4]Department of Environmental Science, Faculty of Veterinary Medicine, Hokkaido University, Hokkaido, Japan

[5]Aoyama Allergy Clinic, Gunma, Japan

[6]Department of Anesthesiology, Tokyo Women's Medical University Medical Center East, Tokyo, Japan

S1 Table. The names, common names, alternative abbreviations in literature and oral toxicity of seven neonicotinoid insecticides and metabolites (This table was originally published by PLoS One 2013; 8: e80332; doi: 10.1371/journal.)

Name used in this study	Common name	Alternative abbreviation**	Oral LD50 (mg/kg BW) in rodents (male)***	Oral LD50 (mg/kg BW) in rodents (female)***
Acetamiprid	Acetamiprid	ACE	198-217	146-184
AM-1	*N*-((6-Chloropyridin-3-yl)-methyl)-*N*-methyl-acetamide	ACE-acet	1142	900-1000
AM-2	*N*-Desmethyl-acetamiprid	ACE-dm	2543	1276
AM-3	*N*-((6-Chloropyridin-3-yl)-methyl)- acetamide	ACE-dm-acet	1378	900-1000
AM-4		ACE-dm-NCONH2		
AM-5		ACE-U		
AM-6	*N*-((6-Chloropyridin-3-yl)-methyl)-methylamine	CNCP-r	1224-1259	963-1176
AM-7	*N*-(6-Chloropyridin-3-yl)-methylamine	CNCP-s	1592	1381
AM-8	*N*-(6-Chloropyridin-3-yl)-formamide	CNCP-t		
AM-9	*N*-Cyano-*N*'-methyl-acetamidine	CNCP-u	2662	2420
AM-10		CNCP-v		
AM-11	*N*-Cyano-acetamidine	CNCP-w	>5000	>5000
AM-12	*N*-Decyano-acetamiprid	ACE-NH	141-195	132-200
AM-13		ACE-$NCONH_2$		
Imidacloprid	Imidacloprid	IMI	100-440	98-475
IM-1	5-Hydroxy-imidacloprid	IMI-5-OH		
IM-2		IMI-de		
IM-3	4,5-Dihydroxy-imidacloprid	IMI-diol		
IM-4	*N*-Denitro-imidacloprid	IMI-NH	300	280
IM-5		IMI-NNH2		
IM-6	*N*-((6-Chloropyridin-3-yl)-methyl)-*N*'-nitroso-imidazolidin-2-ylideneamine	IMI-NNO	200-1980	200-3560
IM-7	4,5-Dehydro-imidacloprid	IMI-ole	3500	1100
IM-8		IMI-tri		
IM-9	*N*-((6-Chloropyridin-3-yl)-methyl)-imidazolidinone	IMI-urea	4080	1820
IM-10		CNCP-l		
IM-11		CNCP-m		
IM-12		IMI-5-OH-gluc		
IM-13		IMI-urea-gluc		
IM-14		IMI-urea-gent		
Clothianidin	Clothianidin	CLO	389->5000	465->5000
CM-1	*N*-Desmethyl-clothianidin	CLO-dm		1480
CM-2	*N*-Desmethyl-*N*'-denitro-clothianidin	CLO-dm-NH		
CM-3		CLO-dm-NNH2		
CM-4		CLO-dm-NNO		
CM-5		CLO-dm-tri		
CM-6		CLO-dm-urea		
CM-7	*N*-Denitro-clothianidin	CLO-NH		
CM-8		CLO-NNH2		
CM-9		CLO-NNO		
CM-10		CLO-tri		
CM-11	*N*-(2-Chlorothiazole-5-methyl)-*N*'-methyl-urea	CLO-urea	1420	1280
CM-12	*N*-methyl-*N*'-nitroguanidine	NG-E		
CM-13	*N*-methyl-guanidine	NG-F	550	446
CPM-1	6-Chloronicotinic aldehyde	CNCP-a		
CPM-2	6-Chloropyridine-3-methanol	CNCP-b (CPOL)	1842, 3800	1483, 3700
CPM-3	6-Chloronicotinic acid	CNCP-c (CPCA)	>5000	>5000
CPM-4		CNCP-d		
CPM-5	2-Methylsulfinylpyrydin-5-yl-carboxilic acid	CNCP-e		
CPM-6		CNCP-f		
CPM-7	2-Hydroxypyridin-5-yl-carboxylic acid	CNCP-g (6-OH-PCA)		
CPM-8	*N*-(6-Chloronicotinoyl)-glycine	CNCP-h		
CPM-9		CNCP-i		
CPM-10		CNCP-j		
CPM-11	Methyl 6-chloronicotinate	CPCA-Me		
CPM-12	2-Mercaptopyrydin-5-yl-carboxilic acid	6-HS-PCA		
CPM-13	2-Pyridone	pyridone		
CPM-14	*N*-(6-Chloronicotinoyl)-alanine	CPCA-Ala		
CPM-15	*N*-(6-Chloronicotinoyl)-aspartic acid	CPCA-ASP		
CPM-16	*N*-(6-Chloronicotinoyl)-cysteine	CPCA-Cys		
CPM-17	*N*-(6-Chloronicotinoyl)-leucine	CPCA-Leu		
CPM-18	*N*-(6-Chloronicotinoyl)-phenylalanine	CPCA-Phe		
CPM-19	*N*-(6-Chloronicotinoyl)-proline	CPCA-Pro		
CPM-20	*N*-(6-Chloronicotinoyl)-threonine	CPCA-Thr		
CPM-21	*N*-(6-Chloronicotinoyl)-valine	CPCA-Val		

CPM-22	*N*-(2-Hydroxypyridin-5-yl-carboxyl)-aspartic acid	6-OH-PCA-Asp		
ICM-1	Nitroguanidine	CNCP-k (NG-G) *	3120-10200	3120-10200
CTM-1	2-Chlorothiazole-5-carboxaldehyde	CTM-a		
CTM-2	2-Chlorothiazole-5-methanol	CTM-b		
CTM-3	2-Chlorothiazole-5-carboxilic acid	CTM-c (CTCA)		
CTM-4		CTM-d		
CTM-5		CTM-e		
CTM-6	*N*-2-Methylsulfinylthiazole-5-carboxylic acid	CTM-f		
CTM-7	*N*-(2-Chlorothiazole-5-carboxyl)-glycine	CTM-g		
CTM-8	*N*-(2-(Methylsulfinyl)thiazole-5-carboxyl)-glycine	CTM-h		
CTM-9	2-Chlorothiazole-5-methylamine	CTM-i		
CTM-10	*N*-Acetyl-2-chlorothiazole-5-methylamine	CTM-j		
Thiacloprid	Thiacloprid	THI	127-836	147-444
	N-Decyano-thiacloprid	THI-NH		1.1-28
	4,5-Dehydro-thiacloprid	THI-ole-NH		
	4-Hydroxy- thiacloprid	THI-4-OH		
		THI-$NCONH_2$		
		THI-4-OH-NCONH2		
		THI-SO		
		THI-SO_3H-$NCONH_2$		
		THI-SOMe		
Nitenpyram	Nitenpyram	NIT	867-1680	1281-1575
	N-Desmethyl-nitenpyram	NIT-dm		
		NIT-dm-COOH		
		NIT-CN		
		NIT-dm-de		
		NIT-desCN		
Thiamethoxam	Thiamethoxam	TMX	783-1563	964-1563
		TMX-NNO		
		TMX-NNH_2		
	N-Denitro-thiamethoxam	TMH-NH		
		TMX-urea		
		NG-A		
		NG-B		
	N-Desmethyl-thiamethoxam	TMX-dm		
		TMX-dm-NNO		
		TMX-dm-NNH_2		
		TMX-dm-tri		
	N-Desmethyl-*N'*-denitro-thiamethoxam	TMX-dm-NH		
		TMX-dm-urea		
		NG-C		
		NG-D		
Dinotefuran	Dinotefuran	DIN	2450-2804	2000-2275
		DIN-NNO		
		DIN-NNH_2		
		DIN-tri		
	N-Denitro-dinotefuran	DIN-NH		
	N-(3-Furayl)-*N'*-methylurea	DIN-urea		
	N-Desmethyl-dinotefuran	DIN-dm		
		DIN-dm-NNO		
		DIN-dm-NNH_2		
		DIN-dm-tri		
	N-Desmethyl-*N'*-denitro-dinotefuran	DIN-dm-NH		
	2-Hydroxy-dinotefuran	DIN-2-OH		
		DIN-a		
		DIN-b		
		DIN-c		
		DIN-d		
		DIN-e		
		DIN-f		
		DIN-4-OH		
		DIN-5-OH		
		DIN-g		
		DIN-h		
		DIN-i		
		DIN-j		
		DIN-k		
	3-Furfural	DIN-l		
	3-Furfuryl alcohol	DIN-m (THFOL)		
	Tetrahydrofuran-3-carboxylic acid	DIN-n (THFCA)		
	N-(Tetrahydrofuran-3-carboxyl)-glycine	DIN-o		
	4-Hydroxy-tetrahydrofuran-3-carboxylic acid	DIN-p		
	N-(4-Hydroxy-tetrahydrofuran-3-carboxyl)-	DIN-q		

	glycine			
	Tetrahydrofuran-3-yl-methylamine	DIN-r (THFMA)		
	N-(Acetyl-tetrahydrofuran-3-yl-methylamine	DIN-s		

*: 2-Nitroguanidine (CNCP-k) and 1-nitroguanidine (NG-G) are tautomeric forms of nitroguanidine and freely convert to each other in solutions.

**: Reference, Ford KA, Casida JE (2006a) Unique and common metabolites of thiamethoxam, clothianidin, and dinotefuran in mice. Chem Res Toxicol 19(11): 1549-56; Ford KA, Casida JE (2006b) Chloropyridinyl neonicotinoid insecticides: diverse molecular substituents contribute to facile metabolism in mice. Chem Res Toxicol 19(7): 944-51; Ford KA, Casida JE (2008) Comparative Metabolism and Pharmacokinetics of Seven Neonicotinoid Insecticides in Spinach. J. Agric. Food Chem. 56(21):10168-75.

***: Reference, Food Safety Commission of Japan. (2008) Pesticide risk assessment, Thiamethoxam. pp17 , *in Japanese*; Food Safety Commission of Japan. (2010) Pesticide risk assessment, Imidacloprid. pp28-30 , *in Japanese*; Food Safety Commission of Japan. (2008) Pesticide risk assessment, Clothianidin. pp 16-17, *in Japanese*; The Health Canada Pest Management Regulatory Agency. (2010) Proposed Registration Decision PRD2010-02, Acetamiprid. 8 February 2010. ISBN: 978-1-100-14565-5 (PDF version). pp37, Available: http://publications.gc.ca/site/eng/363584/publication.html. Accessed 9 April 2013.; Food Safety Commission of Japan. (2005) Pesticide risk assessment, Dinotefuran. pp28-30 , *in Japanese*; Japan Plant Protection Association (2011) Handbook of Pesticide, Japan Plant Protection Association, 2011, pp 65-75, *in Japanese*
