# Supplementary material for: Relationship between Urinary N-Desmethyl-Acetamiprid and Typical Symptoms including Neurological Findings: A Prevalence Case-Control Study
Source: PLoS One. 2015 Nov 4;10(11):e0142172. doi: 10.1371/journal.pone.0142172 (PMC4633099; doi:10.1371/journal.pone.0142172)
Supplement: S2 Table — (PDF) [file pone.0142172.s007.pdf]

Supporting Information

Relationship between urinary *N*-desmethyl-acetamiprid and typical symptoms including neurological findings: A prevalence case-control study

Jemima Tiwaa Marfo[1], Kazutoshi Fujioka[2], Yoshinori Ikenaka[1, 3], Shouta M. M. Nakayama[1], Hazuki Mizukawa[4], Yoshiko Aoyama[5], Mayumi Ishizuka[1], Kumiko Taira[6*]

[1] Laboratory of Toxicology, Department of Environmental Science, Faculty of Veterinary Medicine, Hokkaido University, Hokkaido, Japan

[2]Hawaii Institute of Molecular Education, Hawaii, US

[3]Water Research Group, School of Environmental Sciences and Development, North-West University, South Africa

[4]Department of Environmental Science, Faculty of Veterinary Medicine, Hokkaido University, Hokkaido, Japan

[5]Aoyama Allergy Clinic, Gunma, Japan

[6]Department of Anesthesiology, Tokyo Women's Medical University Medical Center East, Tokyo, Japan

S2 Table. Maximum residue levels (MRLs) of neonicotinoid insecticides in Japan, in compared to EU, US, and CODEX.

Tea leaves

	JPN	EU	US	CODEX
**Acetamprid**	30	0.05*	50**	-
**Clothianidin**	50	0.7	70**	0.7
**Imidacloprid**	10	0.05*	-	-
**Nitenpyram**	10	-	-	-
**Thiacloprid**	30	10	-	-
**Thiamethoxam**	20	20	20**	20
**Dinotefuran**	25	-	50**	-

* indicates lower limit of analytical determination.; **US import tolerance.

Grape, table

	JPN	EU	US	CODEX
**Acetamprid**	5	0.5	0.35	0.5
**Clothianidin**	5	0.7	0.6	0.7
**Imidacloprid**	3	1	1	1
**Nitenpyram**	5	-	-	-
**Thiacloprid**	5	0.02*	-	-
**Thiamethoxam**	2	0.9	0.2	0.5
**Dinotefuran**	15	0.9	0.9	0.9

* indicates lower limit of analytical determination.

Strawberry

	JPN	EU	US	CODEX
**Acetamprid**	3	0.5	0.6	0.5
**Clothianidin**	0.7	0.02*	-	0.07
**Imidacloprid**	0.5	0.5	0.5	0.5
**Nitenpyram**	5	-	-	-
**Thiacloprid**	5	1.0	-	1
**Thiamethoxam**	2	0.5	0.3	0.5
**Dinotefuran**	2	-	-	-

* indicates lower limit of analytical determination.

Pear

	JPN	EU	US	CODEX
**Acetamprid**	2	0.8	1	0.8
**Clothianidin**	1	0.4	1	0.4
**Imidacloprid**	0.7	0.5	0.6	1
**Nitenpyram**	0.5	-	-	-
**Thiacloprid**	2	0.3	0.3	0.7
**Thiamethoxam**	1	0.5	0.2	0.3
**Dinotefuran**	1	-	2***	-

***Expires 12/31/2015.

Peach

	JPN	EU	US	CODEX
**Acetamprid**	2	0.8	1.2	0.7
**Clothianidin**	0.7	0.1	0.8	0.2
**Imidacloprid**	0.5	0.5	3	0.5
**Nitenpyram**	0.5	-	-	-
**Thiacloprid**	1	0.3	0.5	0.5
**Thiamethoxam**	0.5	0.3	0.5	1
**Dinotefuran**	3	0.8	2*	0.8

***Expires 12/31/2015.

Apple

	JPN	EU	US	CODEX
**Acetamprid**	2	0.8	1	0.8
**Clothianidin**	1	0.4	1	0.4
**Imidacloprid**	0.5	0.5	0.5	0.5
**Nitenpyram**	0.5	-	-	-
**Thiacloprid**	2	0.3	0.3	0.7
**Thiamethoxam**	0.3	0.5	0.2	0.3
**Dinotefuran**	2	-	2***	-

***Expires 12/31/2015.

Mandarin, Satsuma (Unsyu)

	JPN	EU	US	CODEX
**Acetamprid**	0.5	0.9	1	1
**Clothianidin**	1	0.1	0.07****	0.07
**Imidacloprid**	0.3	1	0.7	1
**Nitenpyram**	0.5	-	-	-
**Thiacloprid**	5	0.02*	-	-
**Thiamethoxam**	0.3	0.2	0.4	0.5
**Dinotefuran**	2	-	-	-

* indicates lower limit of analytical determination.; ****Expires 12/31/2017.

Cucumber

	JPN	EU	US	CODEX
**Acetamprid**	2	0.3	0.5	0.2
**Clothianidin**	2	0.02*	0.06	0.02
**Imidacloprid**	1	1	0.5	1
**Nitenpyram**	5	-	-	-
**Thiacloprid**	1	0.3	-	0.3
**Thiamethoxam**	0.5	0.5	0.2	0.5
**Dinotefuran**	2	-	0.5	0.5

* indicates lower limit of analytical determination.

Cabbage

	JPN	EU	US	CODEX
**Acetamprid**	3	0.7	1.2	0.7
**Clothianidin**	0.7	0.02*	1.9	0.2
**Imidacloprid**	0.5	0.5	3.5	0.5
**Nitenpyram**	0.03	-	-	-
**Thiacloprid**	1	0.2	-	-
**Thiamethoxam**	5	5	4.5	5
**Dinotefuran**	2	-	1.4	2

* indicates lower limit of analytical determination.

Reference: Codex Pesticide Residues in Food Online Database accessed 2015/09/24; EU Pesticides database accessed 2015/09/24; Global MRL Database^{TM}, A service of Bryant

Christie INC. accessed 2015/09/29; The Japan Food Chemical Research Foundation MRLs List accessed 2015/09/24
