# Supplementary material for: Relationship between Urinary N-Desmethyl-Acetamiprid and Typical Symptoms including Neurological Findings: A Prevalence Case-Control Study
Source: PLoS One. 2015 Nov 4;10(11):e0142172. doi: 10.1371/journal.pone.0142172 (PMC4633099; doi:10.1371/journal.pone.0142172)
Supplement: S3 Table — (PDF) [file pone.0142172.s008.pdf]

Supporting Information

Relationship between urinary *N*-desmethyl-acetamiprid and typical symptoms including neurological findings: A prevalence case-control study

Jemima Tiwaa Marfo[1], Kazutoshi Fujioka[2], Yoshinori Ikenaka[1, 3], Shouta M. M. Nakayama[1], Hazuki Mizukawa[4], Yoshiko Aoyama[5], Mayumi Ishizuka[1], Kumiko Taira[6*]

[1] Laboratory of Toxicology, Department of Environmental Science, Faculty of Veterinary Medicine, Hokkaido University, Hokkaido, Japan

[2]Hawaii Institute of Molecular Education, Hawaii, US

[3]Water Research Group, School of Environmental Sciences and Development, North-West University, South Africa

[4]Department of Environmental Science, Faculty of Veterinary Medicine, Hokkaido University, Hokkaido, Japan

[5]Aoyama Allergy Clinic, Gunma, Japan

[6]Department of Anesthesiology, Tokyo Women's Medical University Medical Center East, Tokyo, Japan

S3 Table. Physicochemical characteristics of neonicotinoid insecticides.

				Solubility (g/L)		
	**MW**	**pK_a of conjugate acid**	**pK_a**	**Water (pH 7)**	**Octanol**	**Acetone**
**Thiamethoxam**	291.7	N/A	N/A	4.1	0.62	48
**Clothianidin**	249.7	N/A	11.1	0.327	1.64	15.2
**Acetamiprid**	222.7	0.7	N/A	2.95	18.5	>200
**Imidacloprid**	255.7	1.56	11.1	0.48	0.78	47
**Thiacloprid**	252.7	N/A	N/A	0.185	3.36	64
**Nitenpyram**	270.7	3.1	11.5	840	192	290
**Dinotefuran**	202.2	N/A	12.6	40	-	58
**Sulfoxaflor**	277.3	N/A	N/A	0.568	1.66	217

Note: pKa stands for the acid dissociation constant of the molecule (M) or the conjugated acid (MH^+). N/A stands for not available. Although pKa of conjugated acid are not known for thiamethoxam, clothianidin or thiacloprid, they theoretically exist because pseudo-molecule ions [MH^+] of those NN are monitored in the positive mode by electrospray ionization mass spectrometry.

Reference:

US Environmental Protection Agency (USEPA) Factsheet-Acetamiprid. 2002.
National Pesticide Information Center. 1.800.858.7378. Imidacloprid Technical Fact Sheet
A Rule by the US Environmental Protection Agency (USEPA) on 03/02/2012.
Thiamethoxam; Pesticide Tolerance. Fed Regist 78:18512-18513
US Environmental Protection Agency (USEPA) Factsheet-clothianidin.2003.
US Environmental Protection Agency (USEPA) Factsheet-thiacloprid. 2003.
US Environmental Protection Agency (USEPA) Factsheet-dinotefuran. 2004.
