# Supplementary material for: Relationship between Urinary N-Desmethyl-Acetamiprid and Typical Symptoms including Neurological Findings: A Prevalence Case-Control Study
Source: PLoS One. 2015 Nov 4;10(11):e0142172. doi: 10.1371/journal.pone.0142172 (PMC4633099; doi:10.1371/journal.pone.0142172)
Supplement: S4 Table — (PDF) [file pone.0142172.s009.pdf]

Supporting Information

Relationship between urinary *N*-desmethyl-acetamiprid and typical symptoms including neurological findings: A prevalence case-control study

Jemima Tiwaa Marfo[1], Kazutoshi Fujioka[2], Yoshinori Ikenaka[1, 3], Shouta M. M. Nakayama[1], Hazuki Mizukawa[4], Yoshiko Aoyama[5], Mayumi Ishizuka[1], Kumiko Taira[6*]

[1] Laboratory of Toxicology, Department of Environmental Science, Faculty of Veterinary Medicine, Hokkaido University, Hokkaido, Japan

[2]Hawaii Institute of Molecular Education, Hawaii, US

[3]Water Research Group, School of Environmental Sciences and Development, North-West University, South Africa

[4]Department of Environmental Science, Faculty of Veterinary Medicine, Hokkaido University, Hokkaido, Japan

[5]Aoyama Allergy Clinic, Gunma, Japan

[6]Department of Anesthesiology, Tokyo Women's Medical University Medical Center East, Tokyo, Japan

S4 Table. Residual Radioactivity of neonicotinoid in Tissues and Organs of Male and Female Rats after a Single Oral Administration of radiolabeled each neonicotinoid.

S4-1. 96 hours after a single oral 1mg/kg Administration, ^{14}C-labeled acetemiprid. (unit: μg equivalent of acetamiprid/kg of tissue (nM equivalent of acetemiprid/kg of tissue))

	**Male**	**Female**
**Spleen**	3 (13.5)	3 (13.5)
**Heart**	6 (26.9)	6 (26.9)
**Bone**	2 (9.0)	2 (9.0)
**Lung**	11 (49.4)	13 (58.4)
**Adrenal gland**	7 (31.4)	4 (18.0)
**Ovary**	No sample	2 (9.0)
**Uterus**	No sample	No data
**Sciatic nerve**	7 (31.4)	1 (4.5)
**Pancreas**	2 (9.0)	2 (9.0)
**Thyroid gland**	5 (22.5)	4 (18.0)
**Whole blood**	8 (35.9)	8 (35.9)
**Brain**	2 (9.0)	2 (9.0)
**Liver**	24 (107.8)	20 (89.8)
**Kidney**	14 (62.9)	13 (58.4)
**Skeletal muscle**	4 (18.0)	4 (18.0)
**Fat**	1 (4.5)	2 (9.0)
**Testis**	1 (4.5)	No sample
**Skin**	6 (26.9)	22 (98.8)

Data are mean of 5 Male rats (mean body weight 181g) and 5 female rats (mean body weight 145g). Reference: Nippon Soda Co., Ltd. Noyaku Shoroku 5/5 p483, in website of Independent Administrative Institution of Food and Agricultural Materials Inspection Center. [in Japanese] Accessed 2015.6.6.

www.acis.fanic.go.jp/shoroku/acetamiprid/adeamiprid_05.pdf

S4-2. 24 hours after a Single Oral 0.5mg/kg Administration, two kind of radioisotope labeled thiamethoxam. (unit: µg equivalent of thiamethoxam/kg of tissue (nM equivalent of thiamethoxam/kg of tissue))

**radioisotope-labeled thiamethoxam**	**1**		**2**	
	**Male**	**Female**	**Male**	**Female**
**Spleen**	1.6 (5.5),	2.1 (7.2)	2.8 (9.6)	1.0 (3.4)
**Heart**	1.3 (4.5)	1.4 (4.8)	5.3 (18.2)	1.3 (4.5)
**Bone**	1.9 (6.5)	1.2 (4.1)	2.6 (8.9)	1.1 (3.8)
**Lung**	1.9 (6.5)	1.7 (5.8)	4.1 (14.1)	1.8 (6.2)
**Ovary**	No sample	5.2 (17.8)	No sample	3.3 (11.3)
**Uterus**	No sample	2.8 (9.6)	No sample	1.8 (6.2)
**Sciatic nerve**	No data	No data	No data	No data
**Pancreas**	No data	No data	No data	No data
**Thyroid gland**	No data	No data	No data	No data
**Whole blood**	2.1 (7.2)	2.6 (8.9)	3.9(13.4)	1.9 (6.5)
**Plasma**	1.7 (5.8)	1.8 (6.2)	3.7 (12.7)	1.6 (5.5)
**Brain**	=LQ (=LQ)	0.8 (2.7)	3.6 (12.3)	0.6 (2.1)
**Liver**	7.0 (24.0)	5.1 (17.5)	14.8 (50.7)	5.6 (19.2)
**Kidney**	4.5 (15.4)	4.7 (16.1)	12.7 (43.5)	2.8 (3.4)
**Skeletal muscle**	1.9 (6.5)	1.2 (4.1)	5.2 (17.8)	1.0 (1.7)
**Fat**	0.6 (2.1)	=LQ (=LQ)	3.3 (11.3)	0.5 (1.7)
**Testis**	1.3 (4.5)	No sample	5.2 (17.8)	No sample
**Skin**	No data	No data	No data	No data

LQ: limit of quantification. Data are mean of 12 rats. Reference: Syngenta Co. Ltd. Noyaku Shoroku 5/6 m-29, 32, in website of Independent Administrative Institution of Food and Agricultural Materials Inspection Center. [in Japanese] Accessed 2015.6.6. www.acis.fanic.go.jp/shoroku/thiamethoxam/thiamethoxam_05.pdf

S4-3. 24 hours after a Single Oral 5 mg/kg Administration of [nitroimino-^{14}C] Clothianidin (unit: μg equivalent of acetamiprid/kg of tissue (nM equivalent of acetemiprid/kg of tissue))

	**[nitroimino-^{14}C]Clothianidin**		**[thiazolyl-2-^{14}C]Clothianidin**	
	**Male**	**Female**	**Male**	**Female**
**Spleen**	30 (120),	70 (280)	40 (160)	40 (160)
**Heart**	30 (120)	100 (400)	40 (160)	50 (160)
**Bone**	No data	No data	No data	No data
**Lung**	30 (120)	80 (320)	40 (160)	50 (200)
**Ovary**	No sample	50 (200)	No sample	30 (120)
**Uterus**	No sample	50 (200)	No sample	30 (120)
**Sciatic nerve**	20 (80)	60 (240)	<10 (<40)	10 (40)
**Pancreas**	20 (80)	60 (240)	30 (120)	30 (120)
**Thyroid gland**	20 (80)	50 (200)	20 (80)	20 (80)
**Whole blood**	30 (120)	80 (320)	70 (280)	90 (360)
**Plasma**	No data	No data	No data	No data
**Brain**	10 (40)	30 (120)	<10 (<40)	10 (40)
**Liver**	140 (561)	240 (961)	180 (721)	170 (681)
**Kidney**	90 (360)	180 (721)	250 (1001)	310 (1241)
**Skeletal muscle**	20 (80)	60 (240)	20 (80)	30 (120)
**Fat**	<10 (<40)	20 (80)	10 (40)	<10(<40)
**Testis**	20 (80)	No sample	30 (120)	No sample
**Skin**	50 (200)	100 (400)	50 (200)	40 (160)
**Adrenal**	40 (160)	180 (721)	50 (200)	60 (240)
**Cecum**	140 (561)	170 (681)	90 (360)	60 (240)
**Intestine**	40 (160)	50 (200)	30 (120)	40 (160)
**Spinal cord**	10 (40)	20 (80)	<10 (<40)	10 (40)
**Stomach**	30 (120)	90 (360)	80 (320)	10 (40)

Data are the mean of three rats. Reference: Yokota T, Mikata K, Nagasaki H, Ohta K. Absorption, Tissue Distribution, Excretion, and Metabolism of Clothianidin in Rats. J. Agric. Food Chem. 2003, 51, 7066−7072
