# Supplementary material for: Relationship between Urinary N-Desmethyl-Acetamiprid and Typical Symptoms including Neurological Findings: A Prevalence Case-Control Study
Source: PLoS One. 2015 Nov 4;10(11):e0142172. doi: 10.1371/journal.pone.0142172 (PMC4633099; doi:10.1371/journal.pone.0142172)
Supplement: S5 Table — (PDF) [file pone.0142172.s010.pdf]

Supporting Information

Relationship between urinary *N*-desmethyl-acetamiprid and typical symptoms including neurological findings: A prevalence case-control study

Jemima Tiwaa Marfo[1], Kazutoshi Fujioka[2], Yoshinori Ikenaka[1, 3], Shouta M. M. Nakayama[1], Hazuki Mizukawa[4], Yoshiko Aoyama[5], Mayumi Ishizuka[1], Kumiko Taira[6*]

[1] Laboratory of Toxicology, Department of Environmental Science, Faculty of Veterinary Medicine, Hokkaido University, Hokkaido, Japan

[2]Hawaii Institute of Molecular Education, Hawaii, US

[3]Water Research Group, School of Environmental Sciences and Development, North-West University, South Africa

[4]Department of Environmental Science, Faculty of Veterinary Medicine, Hokkaido University, Hokkaido, Japan

[5]Aoyama Allergy Clinic, Gunma, Japan

[6]Department of Anesthesiology, Tokyo Women's Medical University Medical Center East, Tokyo, Japan

S5 Table. Case reports analysis of acute imidacloprid and acetamiprid intoxication, and those toxic doses.

S5-1. Case reports of acute imidacloprid intoxication

Author (year)	n	Age/sex	Product Concen -tration (%)	Estimated amount of intake (mL)	Estimated intake (mg/kgBW)	Blood concentration	Product name	*
Mild to moderate cases								
Phua (2009)	40			5-300	8-480			
	12							1
Chwaluk (2010)		48/F		Unknown				8
Severe cases								
Wu (2001)		64/M	9.6	100	160		Tie-Boo-Tzang	2
Tamura（2002）		50/M		25		15 ng/mL		
		95/M	2	25		3 ng/mL		
		78/M	10	unknown		84.9 ng/mL		
		71/M	10	unknown				
		62/M	20	unknown		30.7 ng/mL		
		89/F	10	unknown		23 ng/mL		
Hung (2006)		71/M	9.6	200	320			2
David (2007)		22/M	17.8	30	89			
Agarwal (2007)		24/M	17.8	unknown			Crop King	3
Paningrahi (2009)		37/M	17.8	50	150		OMIDA(SL)	
Karatas (2009)		67/M		unknown			Confidor SC350	
Phua(2009)	8			30-200	48-320			
Viradiya (2011)		41/M	70	75	875			
Lin (2012)		56/M	9.6	40	64		Tie-Sha-Zhan	4
Agha (2012)		62/M	30	Unknown			SUREKILL	7
Lethal cases								
Proensa (2005)		66/M		150	240	2.05μg/mL	Confidor	
		33/M		200	320	12.5μg/mL		5
Huang(2006)		69/F	9.6	200	384		Confidor	2
		64/F	9.6	150	288			2
Shadnia (2008)		36/M		350	395			
Phua (2009)		65/M		50	80			
		84/M		200	320			
Iyyadurai (2010)		34/M						
Yeh (2010)		67/M	18.2	unknown			Cheminova	6
Fuke (2013)		70/M	20.0			105 μg/mL	AdmireFlowable	9

*1: Cases except by oral exposure.
*2: The product contained N-methylpyrrolidone and 2% surfactant.
*3: Occupational inhalational and dermal exposure case.
*4: The product contained N-methylpyrrolidon
*5: 0.18g/L of ethanol was also detected.
*6: The product was ingested with liquor.
*7: occupational exposure case.
*8: inhaled exposure case.
*9：in cerebrospinal fluid, 58.5μg/mL of imidacloprid was detected.

S5-2. Case reports of acute and subacute acetamiprid intoxication

Author (year)	Age /sex	Product Concen -tration (%)	Estimated amount of intake (mL)	Estimated intake (mg/kgBW)	Blood concent-ration (µg/mL)	Product name	*
Mild to moderate case							
Taira (2013)	22/F				3.2ng/mL		5
Severe cases							
Imamura (2010)	58/M	18	18	30	2.39µg/mL	Mospiran SL	1,2
	74/F	2	100	40	59.83µg/mL	Mospiran solution	3
Todani (2008)	79/M	20	35	140	21.1µg/mL	Mospiran water soluble powder	4
Takano（2011）	unknown	2	100	33		Mospiran solution	3
	unknown	2	600	200		Mospiran solution	3
	unknown	18	150	450		Mospiran SL	2
	unknown	18	200	600		Mospiran SL	2
Tanaka（2011）	63/M	2	100	33		Mospiran solution	3
Lethal case							
Takano（2011）	unknown	2	100	33		Mospiran	2
Yeter (2014)	7/F,8/M				2.7µg/mL		6

*1. Patient attempted subcutaneous ingestion as well as oral intake.
*2. Mospiran SL contains acetamiprid 18%, N-methylpyrrolidone 31%, dimethyl sulfoxide 3.05%, and surfactant.
*3. Mospiran solution contains acetamiprid 2%, diethyleneglycol 97%, surfactant 1%.
*4. Mospiran water soluble powder contains 2.4% surfactant.
Source: reference 27-30
*5. Urine concentration of desmethyl-acetamiprid
*6：IM-1-2((E)- N2-carbamoyl-N1- [(6-chloro-3-pyr- idyl)methyl]-N1-methylacetamidine) was not detected in blood. Acetamiprid and IM-1-2 was not detected in urine.

S5-3. The symptoms of acute imidacloprid and acetamiprid intoxication

	Imidacloprid			Acetamiprid
	Severe	Lethal	Total	Total
Number of cases (%)	16	7	23	10
Cardiovascular	16(100)	5(71.4)	21(91.3)	8(80.0)
Tachycardia/bradicardia	12/2	4/2	16/4	3/0
Hypertention/hypotention	5/1	2/1	7/2	5/1
Central nervous system	10(60.0)	6(85.7)	16(69.6)	6(60.0)
Low GCS/unconsciousness	6/3	6/3	12/6	2/4
Sleepiness/dizziness	3/1	2/1	5/2	0/1
Convulsion/excitation	3/3	1/0	4/2	2/0
Respiratory	9(53.3)	6(85.7)	15(65.2)	2(20.0)
Dyspnea/tachypnea	5/4	3/1	8/5	1/1
Respiratory arrest	2	3	5	0
Cough/cyanosis	1/1	0/2	1/3	0
Gastrointestinal	9(60.0)	4(57.1)	13(56.5)	5(50.0)
Nausea/vomiting	9	4	13	5
Oral-esophageal-gastric erosion	1	2	3	0
Secretion	6(40.0)	5(71.4)	11(47.8)	2(20.0)
diaphoresis/anhidrosis	3/1	4/0	7/1	0
Excessive discharge of saliva and bronchial secretion/mouth dryness	3/1	2/0	5/1	0/2
Pupil	6(40.0)	2(28.6)	8(34.7)	1(10.0)
Midriasis/miosis	5/1	2/0	7/1	0/1
Abnormal light reflex	1	2	3	0
Body temperature	5(26.7)	2(28.6)	7(30.4)	6(60.0)
Fever/low body temperature	4/1	2/1	6/2	1/5
Skeletal muscle	2(13.3)	2(28.6)	4(17.4)	3(30.0)
Muscle weakness/muscle spasm/high CK	1/1/1	0/0/2	1/1/3	2/1/0
Others				
Metabolic acidosis	1	2	3	5
Leukoclastic vasculitis/renal・hepatic disfunction	1	0	0	0

S5-4. The toxic dose (mg/kg) of imidacloprid and acetemiprid

	Imidacloprid (n=145)	Acetamiprid (n=9)
Minimum lethal dose	80	
Mean lethal dose	310	
Maximum tolerated dose	875	600
Minimum toxic dose	48	30
Rat Oral LD_{50} (male/female)	440/410	217/146
Rat Inhalational LD_{50}	>5320 mg/m^3	>300 mg/m^3
Dermal LD_{50} (rat)	>5000	>2000
Bee LD_{50} (μg/bee)	0.0179	7.07

Reference:

Proença P TH, Castanheira F, Pinheiro J, Monsanto PV, Marques EP, Vieira DN: Two fatal intoxication cases with imidacloprid: LC/MS analysis. Forensic Sci Int 153: 75- 80, 2005

Wu IW, Lin JL, Cheng ET: Acute poisoning with the neonicotinoid insecticide imidacloprid in N-methyl pyrrolidone. J Toxicol Clin Toxicol 39: 617- 621, 2001

Hung YM LS, Chou KJ, Chung HM: Imidacloprid-N- Methyl Pyrrolidone Insecticides Poisoning Mimicking Cholinergic Syndrome. Clin Toxicol 44: 625–783, 2006

Tamura M, Endo Y, Kuroki Y, Ohashi N, Yoshioka T, Sugimoto T. Investigation and case study of Imidacloprid insecticide caused poisoning. Chudoku Kenkyu. 2002 Jul;15(3):309-12.
[Article in Japanese]

Huang NC, Lin SL, Chou CH, Hung YM, Chung HM, Huang ST: Fatal ventricular fibrillation in a patient with acute imidacloprid poisoning. Am J Emerg Med 24: 883- 885, 2006

David D, George IA, Peter JV: Toxicology of the newer neonicotinoid insecticides: imidacloprid poisoning in a human. Clin Toxicol(Phila)45: 485- 486, 2007

Agarwal R, Srinivas R: Severe neuropsychiatric manifestations and rhabdomyolysis in a patient with

imidacloprid poisoning. Am J Emerg Med 25: 844- 845, 2007

Shadnia S, Moghaddam HH: Fatal intoxication with imidacloprid insecticide. Am J Emerg Med 26: 631- 634, 2008

Mohamed F, Gawarammana I, Robertson TA, Roberts MS, Palangasinghe1 C, Zawahir1 S, Jayamanne S, Kandasamy J, Eddleston M, Buckley1 NA, Dawson AH, Roberts DM: Acute human self-poisoning with imidacloprid compound: a neonicotinoid insecticide. PLoS One 4: e5127, 2009

Panigrahi AK, Subrahmanyam DK, Mukku KK: Imidacloprid poisoning: a case report. Am J Emerg Med 27: 256, 2009

Karatas AD: Severe central nervous system depression in a patient with acute imidacloprid poisoning. Am J Emerg Med 27: 1171, 2009

Iyyadurai R, George IA, Peter JV: Imidacloprid poisoning-newer insecticide and fatal toxicity. J Med Toxicol 6:77- 78, 2010

Yeh IJ, Lin TJ, Hwang DY: Acute multiple organ failure with imidacloprid and alcohol ingestion. Am J Emerg Med 28: 255 e251- 253, 2010

Phua DH, Lin CC, Wu ML, Deng OF, Yang CC: Neonicotinoid insecticides: an emerging cause of acute pesticide poisoning. Clin Toxicol(Phila)47: 336- 341, 2009

Viradiya K, Mishra A: Imidacloprid poisoning. J Assoc Physicians India 59: 594- 595, 2011

Todani M1, Kaneko T, Hayashida H, Kaneda K, Tsuruta R, Kasaoka S, Maekawa T. Acute poisoning with neonicotinoid insecticide acetemiprid. Chudoku Kenkyu. 2008 Oct;21(4):387-90. [Article in Japanese]

Japan Plant Protection Association: Handbook of Pesticide, Japan Plant Protection Association, 2011, pp65-75 [Book in Japanese]
