# Supplementary material for: Relationship between Urinary N-Desmethyl-Acetamiprid and Typical Symptoms including Neurological Findings: A Prevalence Case-Control Study
Source: PLoS One. 2015 Nov 4;10(11):e0142172. doi: 10.1371/journal.pone.0142172 (PMC4633099; doi:10.1371/journal.pone.0142172)
Supplement: S6 Table — (PDF) [file pone.0142172.s011.pdf]

Supporting Information

Relationship between urinary *N*-desmethyl-acetamiprid and typical symptoms including neurological findings: A prevalence case-control study

Jemima Tiwaa Marfo[1], Kazutoshi Fujioka[2], Yoshinori Ikenaka[1, 3], Shouta M. M. Nakayama[1], Hazuki Mizukawa[4], Yoshiko Aoyama[5], Mayumi Ishizuka[1], Kumiko Taira[6*]

[1] Laboratory of Toxicology, Department of Environmental Science, Faculty of Veterinary Medicine, Hokkaido University, Hokkaido, Japan

[2]Hawaii Institute of Molecular Education, Hawaii, US

[3]Water Research Group, School of Environmental Sciences and Development, North-West University, South Africa

[4]Department of Environmental Science, Faculty of Veterinary Medicine, Hokkaido University, Hokkaido, Japan

[5]Aoyama Allergy Clinic, Gunma, Japan

[6]Department of Anesthesiology, Tokyo Women's Medical University Medical Center East, Tokyo, Japan

S6 Table. Time course of 6-chrolonicotinic acid (6-CNA) and the maximum 6-CNA concentrations in the urine sample of eleven patients, and their subjective symptoms, clinical findings, electrocardiogram findings, and food/beverage intake.

These tables were firstly published in 2011, Chudoku-Kenkyu 24:22-230 as in part of an original article, Detection of chloropyridinyl neonicotionoid insecticide metabolite 6-chloro-nicotinic acid in the urine: Six cases with subacute nicotinic symptoms. Taira K, Aoyama Y, Kawakami T, Kamata M, and Aoi T.

Summary:
Neonicotinoid is a recently developed insecticide with worldwide use that has been increasing. It acts as a nicotinic acetylcholine receptor agonist. Chloropyridinyl neonicotinoid is a subgroup of neonicotinoid, and are commercially available as imidacloprid, nitenpyram, acetamiprid, and thiacloprid. The maximum residue limits of acetamiprid for fruits and tea leaves are high in Japan, e.g. 5 ppm for grapes and 30 ppm for tea leaves. 6-chloronicotinic acid (6 CNA) is a common metabolite in animals after exposure to chloropyridinyl neonicotinoids, but has not yet been detected in human urine. 'Spot' urine samples on the first visit and after were collected from eleven patients 6-52 years-old, who visited X-clinic from August to December in 2008, within 24 hours after symptom onset with unknown origin. Urinary 6 CNA was detected in six out of the eleven patients (IC positive group), by ion chromatography and identified in twenty specimens of these six patients by liquid chromatography-mass spectrometry (LC/MS), maximum 84.8 microg/L from the first visit to the 20th visit. The sensitivity of ion chromatography for LC/MS was 45%, and the specificity was 100%. The IC positive group showed headache, general fatigue, finger tremor, and short time memory disturbance in 100%, fever (> 37.0 degrees C), cough, palpitation, chest pain, stomachache, myalgia/muscle spasm/muscle weakness in 83%, heart rate abnormality (sinus tachycardia, sinus bradycardia, or intermittent WPW syndrome) in 83%, high domestic fruits intake (> 500 g/day) in 83%, high tea beverage intake (> 500 mL/day) in 66%. Five patients who were not among the IC positive group showed < 80%, < 40%, 60%, 60%, 20%, respectively. The patients gradually recovered through supportive therapy and the restriction of fruits and tea intake within several days to two months. In conclusion, urinary 6-chloronicotinic acid, a common metabolite of chloropyridinyl neonicotinoid insecticide, was detected for the first time, from six patients with subacute nicotinic symptoms.

Table 2 Time course of detection of 6-chloronicotinic acid (6 CNA) and the maximum 6 CNA concentrations (μg/L) in the urine samples of eleven cases

case			the day of illness																		maximum 6 CNA
#	age	sex	1	2	3	4	5	6	7	8	9	10	11	12	13	14	20	26	36	43	(μg/L)
1	34	F	−		−	+	++	+	+					++		++			−	−	12
2	22	F	+	++	−	−	−			++											59.1
3	6	F	+		−												++				16.2
4	45	F	++			−															7.5
5	17	M	+	++																	45.1
6	9	F	+		+		+		+		++										84.8

− : means 6 CNA was not detected by LC/MS or by IC
+ : means 6 CNA was detected by LC/MS but not by IC
++ : means 6 CNA was detected by LC/MS and by IC

Detection limits of 6 CNA by LC/MS and by ion chromatography (IC) are 2 μg/L and 40 μg/L, respectively

Table 4 Comparison of subjective symptoms, clinical findings, electrocardiogram findings, and food/beverage intake between the three groups

group	IC positive	IC negative	chronic
onset of the symptoms	<24 hs	<24 hs	>24 hs
number of cases	6	5	22
6 CNA positive by ion chromatography	6	0	1#
age (mean±SD)	22.2±15.0	32.6±16.2	40.1±20.1
sex (M/F)	1/5	1/4	9/13
subjective symptoms			
headache	6***	3	13
general fatigue	6***	3	17*
palpitation	5**	1	10
chest pain	4**	0	10
stomachache	4**	1	4
myalgia	3	1	8
throat pain	3	2	6
nausea	2	2	10
sleep disturbance	2	1	12
shoulder stiffness	1	3	12
constipation	0	2	8
clinical findings			
finger tremor	6***	4*	12
short time memory disturbance	6***	2	13
JCS Ⅰ-1	6***	2	13
fever (>37.0℃)	5**	1	7
cough	5**	1	10
muscle weakness/spasm	3	2	12
skin eruption	0	2	8
electrocardiogram abnormality	6***	5**	10
rhythm	5**	3*	5
ST change	3	0	7
long QT	1	2	2
food and bevarage			
tea (>500 mL/day)	4*	1	13
fruits (>500 g/day)	5**	3*	17*
vegetable(salad/soup/juice)	2	5**	6

* means more than 60%, ** means more than 80%, and *** means 100%

6 CNA of chronic group were analyzed by ion chromatography only on the first visit
