# Supplementary material for: Relationship between Urinary N-Desmethyl-Acetamiprid and Typical Symptoms including Neurological Findings: A Prevalence Case-Control Study
Source: PLoS One. 2015 Nov 4;10(11):e0142172. doi: 10.1371/journal.pone.0142172 (PMC4633099; doi:10.1371/journal.pone.0142172)
Supplement: S7 Table — (PDF) [file pone.0142172.s012.pdf]

Supporting Information

Relationship between urinary *N*-desmethyl-acetamiprid and typical symptoms including neurological findings: A prevalence case-control study

Jemima Tiwaa Marfo[1], Kazutoshi Fujioka[2], Yoshinori Ikenaka[1, 3], Shouta M. M. Nakayama[1], Hazuki Mizukawa[4], Yoshiko Aoyama[5], Mayumi Ishizuka[1], Kumiko Taira[6*]

[1] Laboratory of Toxicology, Department of Environmental Science, Faculty of Veterinary Medicine, Hokkaido University, Hokkaido, Japan

[2]Hawaii Institute of Molecular Education, Hawaii, US

[3]Water Research Group, School of Environmental Sciences and Development, North-West University, South Africa

[4]Department of Environmental Science, Faculty of Veterinary Medicine, Hokkaido University, Hokkaido, Japan

[5]Aoyama Allergy Clinic, Gunma, Japan

[6]Department of Anesthesiology, Tokyo Women's Medical University Medical Center East, Tokyo, Japan

S7 Table. Urinary creatinine, urinary cystatin C and urinary creatinine / cystatin C ratio (UCCR) in TSG, ASG and NSG.

	TSG	ASG	NSG	p value [a]
**n**	16	15	50	
**Urinary creatinine (mmol/L, median±Q)**	7.62±3.00	3.14±10.18	6.14±1.60	0.153
**Urinary cystatin C (μg/L, median±Q)**	35.0±23.2	4.97±2.25	14.0±16.7	0.382
**UCCR (μg/mmol Cr)**	5.02±1.87	1.80±2.09	2.77±1.09	0.033
**UCCR value (n)**				
**<2**	3	8	15	
**≥2, <4**	3	3	20	
**≥4, <6**	4	0	9	
**≥6, <8**	4	3	2	
**≥8**	2	3	4	
**DMAP detection (n (%))**				
**UCCR value <2**	0 (0)	0 (0)	0 (0)	
**≥2, <4**	2 (66.6)	0 (0)	2 (10.0)	
**≥4, <6**	1 (25.0)	0 (0)	1 (11.1)	
**≥6, <8**	4 (100.0)	0 (0)	0 (0)	
**≥8**	2 (100)	1 (33.3)	0 (0)	
**Thiamethoxam detection (n (%))**				
**UCCR value <2**	0 (0)	0 (0)	0 (0)	
**≥2, <4**	2 (66.6)	0 (0)	0 (0)	
**≥4, <6**	3 (75.0)	0 (0)	0 (0)	
**≥6, <8**	1 (25.0)	0 (0)	0 (0)	
**≥8**	0 (0)	1 (33.3)	0 (0)	
**DMAP or NN detection (n (%))**				
**UCCR value <2**	0 (0)	1 (12.5)	1 (6.7)	
**≥2, <4**	3 (100)	0 (0)	2 (10.0)	
**≥4, <6**	4 (100)	0 (0)	1 (11.1)	
**≥6, <8**	4 (100)	0 (0)	0 (0)	
**≥8**	2 (100)	1 (33.3)	1 (25.0)	

[a] Mann-Whitney test for TSG vs. NSG.
