# Supplementary material for: Relationship between Urinary N-Desmethyl-Acetamiprid and Typical Symptoms including Neurological Findings: A Prevalence Case-Control Study
Source: PLoS One. 2015 Nov 4;10(11):e0142172. doi: 10.1371/journal.pone.0142172 (PMC4633099; doi:10.1371/journal.pone.0142172)
Supplement: S8 Table — (PDF) [file pone.0142172.s013.pdf]

Supporting Information

Relationship between urinary *N*-desmethyl-acetamiprid and typical symptoms including neurological findings: A prevalence case-control study

Jemima Tiwaa Marfo[1], Kazutoshi Fujioka[2], Yoshinori Ikenaka[1, 3], Shouta M. M. Nakayama[1], Hazuki Mizukawa[4], Yoshiko Aoyama[5], Mayumi Ishizuka[1], Kumiko Taira[6*]

[1] Laboratory of Toxicology, Department of Environmental Science, Faculty of Veterinary Medicine, Hokkaido University, Hokkaido, Japan

[2]Hawaii Institute of Molecular Education, Hawaii, US

[3]Water Research Group, School of Environmental Sciences and Development, North-West University, South Africa

[4]Department of Environmental Science, Faculty of Veterinary Medicine, Hokkaido University, Hokkaido, Japan

[5]Aoyama Allergy Clinic, Gunma, Japan

[6]Department of Anesthesiology, Tokyo Women's Medical University Medical Center East, Tokyo, Japan

S8-1. Table. The clinical course of TSG and urinary levels of DMAP and NNs

						Symptoms									Comorbidity	ECG			Urine analysis											
Case No.	Age	Gender	Onset of neo-nicotinic symptoms (days ago)	Number of days for improvement	Food and beverage intake moro than fruits 500g/day or tea beverage 500mL/day suspected	headache	general fatigue	palpitation/chest pains	abdominal pain	muscle symptoms	cough	postural finger tremor	recent memory loss	fever (>37 centigrade)		Sinus tachycardia	ST-T change	others	protein	occult blood	ketone bodies	u-Cre (mmol/L)	UCCR (µg/mmol Cr)	Imidacloprid (nmol/mmol Cr)	Thiacloprid (nmol/mmol Cr)	Nitenpyram (nmol/mmol Cr)	Clothianidin (nmol/mmol Cr)	Acetamiprid (nmol/mmol Cr)	Thiamethoxam (nmol/mmol Cr)	DMAP (nmol/mmol Cr)
1	12	M	U.N.	N.I.	black tea	+	+	+	+	+	-	+	+	+	Psychiatry disease(-3yrs)	-	+		-	-	-	N.E.	N.E.	<LOD	<LOD	<LOD	<LOD	<LOD	<LOD	<LOD
2	16	M	>365	N.I.	black tea	+	+	+	+	+	-	+	+	+	Psichiatry disease	+	+		-	-	-	N.E.	N.E.	<LOD	<LOD	<LOD	<LOD	<LOD	<LOD	<LOD
3	56	F	45	5	green juice, black tea	+	+	+	+	+	+	+	+	-	sleepless	+	+		-	-	-	7.7	8.2	<LOD	<LOD	<LOD	<LOD	<LOD	<LOD	<LOQ
4	62	F	1	N.I.	Japanese pear, peach	+	+	+	+	+	-	+	+	+	low consciousness level JCS-1	-	+		-	-	-	N.D.	N.D.	<LOD	<LOD	<LOD	<LOD	<LOD	<LOD	<LOQ
5	16	M	365	N.I.	unknown	+	+	+	+	+	-	+	+	+	none	+	+		-	-	-	13.8	6.5	<LOD	<LOD	<LOD	<LOD	<LOD	0.31	<LOQ
6	14	F	0.25	U.N.	cucumber, cabbage, other fresh vegitables	+	+	+	+	+	-	+	+	-	low consciousness level JCS-1, invollantary movement	+	+		-	-	-	2.3	1.7	<LOD	<LOD	<LOD	<LOD	<LOD	<LOD	<LOD
7	40	F	425	180	unknown	+	+	+	+	+	-	+	+	+	electromagnetic sensitivity, fiboromyargia	+	+		-	-	-	4.5	2.6	<LOD	<LOD	<LOD	<LOD	<LOD	<LOQ	<LOQ
8	51	F	21	7	vegetable juice	+	+	+	+	+	+	+	+	-	difficuty in writing and stepping, anxiety	-	+		-	-	-	0.7	6.2	<LOD	<LOD	<LOD	<LOD	<LOD	<LOD	<LOQ
9	36	M	1	9	apple, strawberry, orange, green tea, Oolong tea	+	+	+	+	+	+	+	+	+	diabetete mellitus	+	+		-	-	3+	7.6	4.4	<LOD	<LOD	<LOD	<LOD	<LOD	0.44	<LOD
10	13	F	1	U.N.	Oolong tea	+	+	+	+	+	+	+	+	+	none	+	+		-	-	-	8.0	3.7	<LOD	<LOD	<LOD	<LOD	<LOD	0.58	<LOD
11	5	F	U.N.	1	Jasmine tea	+	+	+	+	+	+	+	+	+	none	+	+		-	-	-	5.2	6.9	<LOD	<LOD	<LOD	<LOD	<LOD	<LOD	2.8
12	39	F	>2000	50	Po'er tea	+	+	+	+	+	+	+	+	-	panic syndrome	-	+	a	-	-	-	2.7	0.2	<LOD	<LOD	<LOD	<LOD	<LOD	<LOD	<LOD
13	62	F	>365	21	Oolong tea, vagetables	+	+	+	+	+	-	+	+	-	edema, hyperlipidemia, blood creatine was W.N.L.	-	+		-	-	-	8.3	4.9	<LOD	<LOD	0.54	<LOD	<LOD	0.24	<LOD
14	11	F	14	3	strawberry	+	+	-	+	+	+	+	+	+	nausea	-	+	b	-	-	3+	8.0	6.7	<LOD	<LOD	<LOD	<LOD	<LOD	<LOD	3.6
15	48	M	0.5	U.N.	vegetable salad, punpkin soup	+	+	+	+	+	+	+	+	-	none	-	+	c	-	-	-	26.6	8.1	<LOD	<LOD	<LOQ	<LOD	<LOD	<LOD	<LOD
16	22	F	4	53	Oolong tea, green tea	+	+	+	-	+	+	+	+	+	none	+	+		-	-	-	3.9	5.2	<LOD	<LOD	<LOD	<LOD	<LOD	<LOD	3.0
17	13	M	90	U.N.	green tea, Oolong tea, apple juice	+	+	+	+	+	-	+	+	-	severe migrane with orthostatic dysregulation	+	+		-	-	-	15.7	2.9	<LOD	<LOD	<LOD	<LOD	<LOD	<LOD	<LOQ
18	69	F	1000	203	green juice, apple juice, tea leaves	+	+	+	+	+	-	+	+	-	involuntary movement of head	+	+		-	-	-	1.2	0.2	<LOD	<LOD	<LOD	<LOD	<LOD	<LOD	<LOD
19	49	F	1000	165	unknown	+	+	+	+	+	-	+	+	-	auditory and visual hallucination	-	+	d	-	-	-	15.1	5.1	<LOD	<LOD	<LOD	<LOD	<LOD	<LOQ	<LOD

a: bradycardia, right ventircular electrical conduction delay; b: QT prolongation; c: left bundle brach block; d: bradycardia, QT prolongation

N.I.: not improved; U.N. : unown; N.E.: not examined; JCS: Japan Coma Scale; W.N.L.: with in normal limit; <LOD: less than limit of detetion; <LOQ: less than limit of quantification but more than limit of detection

S8-2. Table. The clinical course of ASG and and urinary levels of DMAP and NNs

						Symptoms									Comorbidity	ECG			Urine analysis											
Case No.	Age	Gender	Onset of neo-nicotinic symptoms (days ago)	Number of days for improvement	Food and beverage intake more than fruits 500g/day or tea beverage 500mL/day suspected	headache	general fatigue	palpitation/chest pains	abdominal pain	muscle symptoms	cough	postural finger tremor	recent memory loss	fever (>37 centigrade)		Sinus tachycardia	ST-T change	others	protein	occult blood	ketone bodies	u-Cre (mmol/L)	UCCR (μg/mmol Cr)	Imidacloprid (nmol/mmol Cr)	Thiacloprid (nmol/mmol Cr)	Nitenpyram (nmol/mmol Cr)	Clothianidin (nmol/mmol Cr)	Acetamiprid (nmol/mmol Cr)	Thiamethoxam (nmol/mmol Cr)	DMAP (nmol/mmol Cr)
1	31	F	24	U.N.	Green tea, apple	+	+	+	+	+	-	+	-	-	none	+	-		-	-	-	N.E.	N.E.	<LOD	<LOD	<LOD	<LOD	<LOD	<LOD	>LOQ
2	31	F	7	U.N.	Japanese pear, peach, grapes	+	+	+	+	+	-	+	-	-	none	+	+		-	-	-	18.9	8.4	<LOD	<LOD	<LOD	<LOQ	<LOD	0.35	<LOQ
3	40	M	U.N.	U.N.	unknown	-	-	-	-	-	-	+	-	-	urticaria	-	-	a	-	-	-	1.6	0.1	<LOD	<LOD	<LOQ	<LOD	<LOD	<LOD	<LOD
4	63	F	1	35	Oolong tea, Japanese pear, gree	+	+	+	-	+	-	+	+	-	none	-	-	b	-	-	-	4.2	3.7	<LOD	<LOD	<LOD	<LOD	<LOD	<LOD	<LOD
5	65	M	20	13	Apple	+	+	-	-	+	-	-	-	-	ESR 101mm/60 min, CRP 5.7	+	-	c	-	-	-	5.1	3.2	<LOD	<LOD	<LOD	<LOD	<LOD	<LOD	<LOD
6	17	F	6	18	tea drinks	+	+	-	+	+	-	+	+	+	ulcerative colitis (10 days after)	-	-		-	-	-	7.1	1.8	<LOD	<LOD	<LOD	<LOD	<LOD	<LOD	<LOD
7	13	M	1500	66	green tea, Japanese pear	+	+	-	+	-	+	+	-	-	Pneumonia	+	-		-	-	-	10.7	8.8	<LOD	<LOD	<LOD	<LOD	<LOD	<LOD	<LOD
8	78	F	21	145	green tea, orange	+	-	-	-	-	-	+	+	-	Kaposi varicelliform eruption, edema, malignant lymphoma	-	-		-	-	-	1.2	1.6	<LOD	<LOD	<LOD	<LOD	<LOD	<LOD	<LOD
9	5	F	21	U.N.	peach, peach juice	+	+	+	+	-	+	+	-	+	Mycoplasma pneumonia (2 days after)	+	-		-	-	-	4.1	6.9	<LOD	<LOD	<LOD	<LOD	<LOD	<LOD	<LOD
10	39	F	U.N.	U.N.	black tea	+	+	+	-	-	-	-	-	-	none	-	-	d	-	-	-	3.1	1.3	<LOD	0.18	<LOQ	<LOD	<LOD	<LOD	<LOD
11	7	F	7	U.N.	black tea	+	+	+	+	-	+	+	+	+	none	-	-		-	-	-	1.3	0.5	<LOD	<LOD	<LOD	<LOD	<LOD	<LOD	<LOD
12	12	M	2	39	green tea, grapes	+	+	-	-	-	+	-	-	-	none	-	-	e	-	-	-	1.3	0.5	<LOD	<LOD	<LOD	<LOD	<LOD	<LOD	<LOD
13	6	F	1	28	grapes	+	+	+	+	-	+	+	+	+	none	-	-	-	-	-	-	2.2	0.9	<LOD	<LOD	<LOD	<LOD	<LOD	<LOD	<LOD
14	11	M	60	U.N.	U.N.	+	+	+	-	-	+	+	-	+	none	-	-	-	-	-	-	2.2	2.2	<LOD	<LOD	<LOD	<LOD	<LOD	<LOD	<LOD
15	47	F	U.N.	28	green tea	+	+	-	-	+	+	+	-	-	Kaposi varicelliform eruption, trichophytia, atypical mycobacteriosis	-	-	d, e	-	-	-	1.6	1.3	<LOD	<LOD	<LOD	<LOD	<LOD	<LOD	<LOD
16	30	M	U.N.	U.N.	U.N.	+	+	+	+	-	-	+	-	-	atopic dermatitis (whole body)	-	-	g	-	-	-	26.9	9.8	<LOD	<LOD	<LOD	<LOD	<LOD	<LOD	<LOD

a: short PR time; b: atrial fibrillation; c: WPW syndrome; d: right ventricular conduction delay; e: junctional rhythm; f: ventricular conduction delay
N.I.: not improved; U.N. : unnown; N.E.: not examined; JCS: Japan Coma Scale; W.N.L.: with in normal limit; <LOD: less than limit of detection;
<LOQ: less than limit of quantification but more than limit of detection
