# Supplementary material for: Relationship between Urinary N-Desmethyl-Acetamiprid and Typical Symptoms including Neurological Findings: A Prevalence Case-Control Study
Source: PLoS One. 2015 Nov 4;10(11):e0142172. doi: 10.1371/journal.pone.0142172 (PMC4633099; doi:10.1371/journal.pone.0142172)
Supplement: S1 Text — (PDF) [file pone.0142172.s014.pdf]

「第22回日本臨床環境医学会学術集会特集」

**Review** Symposium

# Human neonicotinoids exposure in Japan

Kumiko Taira

Department of Anesthesiology, Tokyo Women's Medical University Medical Center East

## Abstract

In Japan, there are currently seven neonicotinoid (NN) insecticides, e.g. acetemiprid, since 1992 registration of imidacloprid. The amount of domestic shipment of NNs was 407t in 2009. NNs are water-soluble, systemic, non-volatile and thermostable. Japanese MRLs for NNs are set at high levels compared to the rest of the world. NNs show persistent efficacy in plants, soil and natural water. Once ingested, NNs are transported through intestinal mucosa, blood-brain-barrier and placenta.

The suspected health effects of environmental exposure to NNs were observed in Japan.

1. Inhalational exposure: 78 patients in 2004 and 63 patients in 2005 visited a clinic in Gunma prefecture with headache, chest pain and muscle pain after acetamiprid spray application to pinewoods for a few weeks.
2. Oral exposure: Since 2006, more than 4,000 patients visited the clinic after consecutive intake of conventional produce, e.g. domestic fruits/vegetables 500 g/day and/or tea beverage 500 mℓ /day, with health problems (neo-Conventional Produce Syndrome, neo-CPS). Urinary 6-chloronicotinic acid (a common metabolite of four NNs) was quantified by liquid chromatography/mass spectrometry at 7.5-84.8 ng/mℓ from 6 neo-CPS patients with finger tremor, impaired short-term memory, fever, general fatigue, headache, palpitation/chest pain, abdominal pain, muscle pain/muscle weaknes/muscle spasm, and cough (neo-nicotinic symptoms), as well as abnormal electrocardiogram.
   From the urine of 2 neo-CPS patients with neo-nicotinic symptoms, acetamiprid and seven NN metabolites were qualitatively detected by liquid chromatography/time-of-flight mass spectrometry; and urinary *N*-desmethyl-acetamiprid (a unique metabolite of acetamiprid) was quantified by liquid chromatography/tandem mass spectrometry at 3.2 ng/m ℓ from one of them.

( Jpn J Clin Ecol 23 : 14－24, 2014)

《**Key words**》 neonicotinoid, insecticide, acetemiprid, imidacloprid, intoxication

## Ⅰ. Introduction

Neonicotinoid (NN) is a systemic insecticide that possesses nicotinic action as an α4β2 nicotinic acetylcholine receptor agonist; and its usage is increasing in the world. The EU decided to ban three NN insecticides for two years starting in 2013 because of their concern about the decline of the honeybee population[1]. In Japan, no valid survey system exists to watch the number of pollinators; however, there are some reports of the decline of the honeybee population[2].

We have been studying the environmental health effects of NN in Gunma prefecture in Japan for ten years[3]. In 2013, a metabolite of NN was quantified

Reprint Requests to Kumiko Taira, Department of Anesthesiology, Tokyo Women's Medical University Medical Centre East, 2-1-10 Nishiogu, Arakawa, Tokyo 116-8567, Japan

from the urine of patients, who were suspected of environmental NN intoxication[4]. Environmental exposure to NN may lead to typical cardiovascular and nervous symptoms, which are distinct from those caused by organophosphates (OPs), in addition to the adverse effects on pollinators.

In this review, "what neonicotinoids are" is discussed first, followed by "case presentation of environmental neonicotinoid exposure" and finally "why neonicotinoid intoxication was observed in Japan".

## Ⅱ. What are neonicotinoids?

As of 2014, seven NNs are commercially available in Japan, namely, imidacloprid, acetamiprid, nitenpyram, thiacloprid, clothianidin, thiamethoxam and dinotefuran (Figure 1). Six out of seven of the NNs possess the same characteristics as chlorinated organic compounds. Chlorinated organic compounds are characterized by their strong and prolonged action, for example organochlorine (OC) insecticide, including DDT. Recently, two novel insecticides with nicotinic action, cycloxaprid and sulfoxaflor, are registered in foreign countries[5, 6].

The shipment of NN in Japan has been increasing. In 1992, imidacloprid was registered first, followed by acetamiprid. In 2012, non-chlorinated NN, dinotefuran, was the most shipped, followed by imidacloprid, clothianidin, and acetemiprid (Figure 2). NNs are used in various situations, e.g. agriculture, home gardening, forestry, lawn, termite extermination, building-products, drugs for animal and disinfectants[7]. In the last ten years, the total shipment of NN insecticides increased more than two-fold, while that of OP insecticides reduced by half to approximately 3,000t, which is comparable to 400t of NNs. NN insecticides are more potent than OP insecticides by weight[7].

The characteristics of three classes of insecticides are summarized in Table1[8–12]. Acetamiprid represents NN; MEP (Fenitrothion) represents OP; and DDT represents OC insecticides. NN is water-soluble and systemic. It penetrates into the plant body and exerts its effects. NN cannot be removed from fruits and vegetables even by washing them with soap water. NN has a very low volatility. However, NN could be disseminated by contaminating in particle matter[13]. NN is long-lasting in efficacy. The efficacy of NN, of

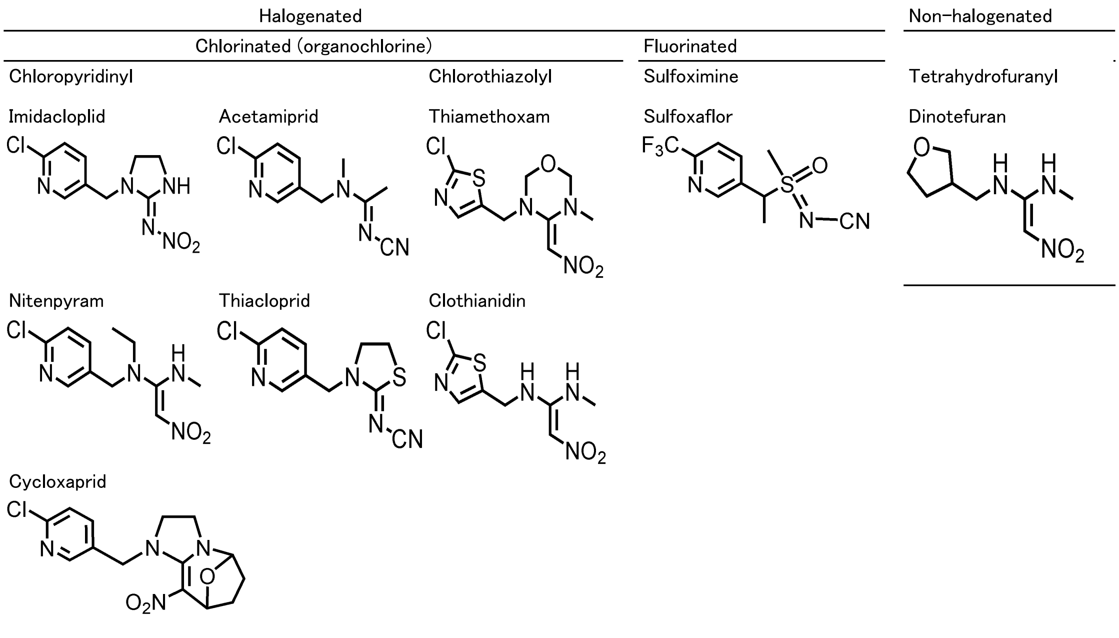


**Figure 1 Chemical structures of neonicotinoid insecticides.**

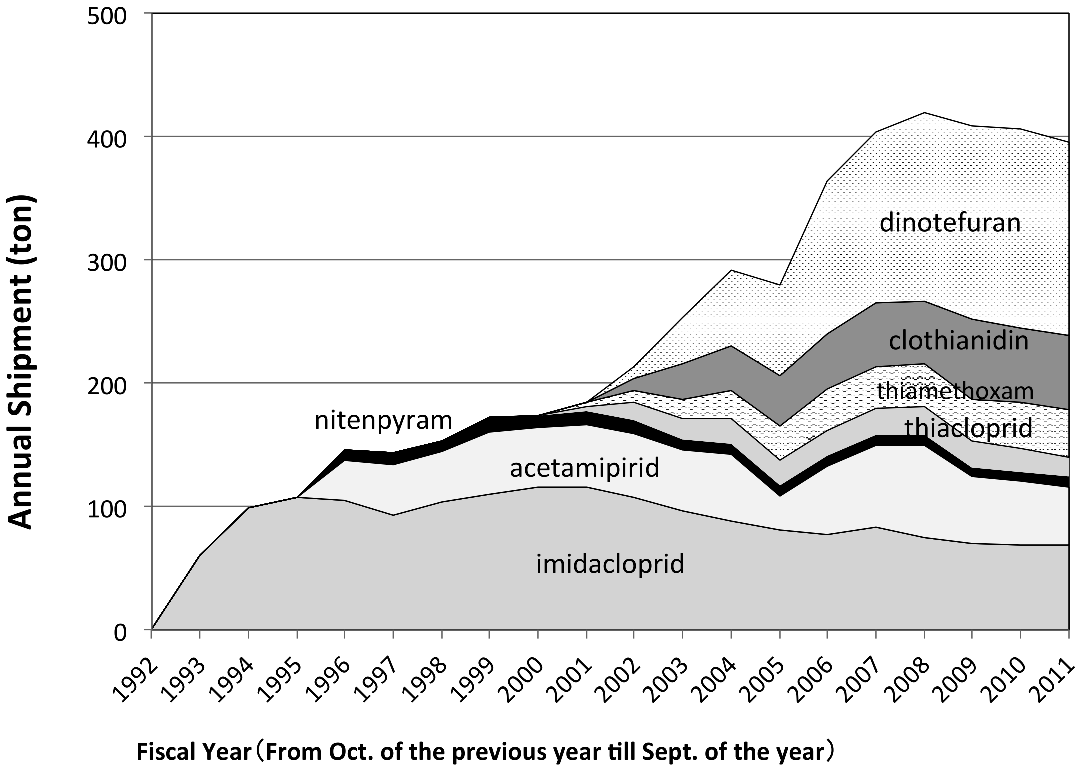


**Figure 2　The shipment of neonicotinoids in Japan.**

(Source: Webkis-plus[29]), Center for Environmental Risk Research, National Institute for Environmental Study)

**Table 1　Characteristics of three classes of insecticides**

| Class (Abbreviation) | Neonicotinoid (NN) | Organophosphate (OP) | Organochlorine (OC) |
|---|---|---|---|
| For example | Acetamiprid | MEP | DDT |
| Water solubility | High | Low | Very low |
| log Pow (20 ℃) | 0.8 | 3.43 | 6.76 |
| Volatility | Very low | Medium | Low |
| Vapor pressure (Pa) | $<1.0\times10^{-6}$ *1 | $1.57\times10^{-3}$ *2 | $2.53\times10^{-5}$ *3 |
| Efficacy period | Long | Short | Very long |
| Half life in water | 349 days *4 | 57days *5 | 150 years |
| Bio-concentration | − | − | + |
| Log BCF | 2 | 1.3-2.6 | 2.7-4.9 |
| Bio-accumulation | Possible | − | + |
| Environmental accumulation | Possible | − | + |
| Toxicity for mammals | | | |
| LD50 (rat♂, mg/kg BW) | 217 | 945 | 113 |
| Neurodevelopmental toxicity | Suspected | Suspected | Suspected |

*1: 25 ℃, *2: 25 ℃, *3: 20 ℃, *4: natural water, *5: pH 7.1, 30 ℃,
Reference: 8-12)

course, is not as long as OC, but longer than OP. For example, the half-life of acetamiprid in natural water is about one year. In soil, more than 3 months are needed for an 80% decline of acetamiprid and its metabolite[9], so environmental accumulation of NN may occur[14]. Bio-concentration factors of NNs are lower than DDT[10, 12] because of its high water-solubility and low octanol-water partition coefficient. Meanwhile how much bioaccumulation of NNs occurs in human body is not known.

## Ⅲ. Environmental neonicotinoid exposure and health effect

### ⅰ. Subacute abnormal electrocardiographic findings observed after acetamiprid spray application for pinewoods

Gunma prefecture is located in the middle of Japan. Two million people live in this 700 $km^2$ basin, at the head of the Tone River.

In the summer of 2004, acetamiprid was sprayed for pinewoods as a countermeasure against pine wilt disease. A 0.02% acetamiprid aqueous solution was applied on pinewood forests in the mountainsides with air blast spraying equipment, which blew the solution to a height of 40 m or higher above the ground during several weeks. Seventy-eight patients visited a clinic in Gunma prefecture 0.5-3 days after the application with symptoms of central nervous system 91% (headache, general fatigue, depression, impairment of concentration, sleep disorders, memory impairment, or fretfulness and difficulty in speech), skeletal muscle 91% (shoulder stiffness, muscle weakness/muscle spasms/muscle pain or tremor), cardiovascular system 77% (chest pain or palpitations), body temperature 77% (fever or coldness of extremities), eye 67.9% (accommodation disorder, photophobia or reduced visual acuity), gastrointestinal tract 60% (abdominal pain, diarrhea or constipation), respiratory system 42.3% (cough or sputum), secretory gland 30.8% (sweating, salivation or thirst/dry mouth) and electrocardiographic abnormalities 89% (ST-T changes 68%, bradycardia, tachycardia, T-wave abnormality or prolonged QT intervals)[7]. Their demographic data are shown in Table 2. During this season, OP (mainly DEP and MEP) was also applied a little.

The following year, acetamiprid was applied again; and sixty-three patients visited the clinic with chest pain, chest discomfort or palpitations with headache, nausea

**Table 2 Demographic data and electrocardiographic findings of patients after the acetemiprid ground application**

| Year | 2004 | 2005 |
|---|---|---|
| Applied pesticide | Acetamiprid (+OP) | Acetamiprid |
| Applied period | 5.26-6.28 | 5.17-6.24 |
| Total spread volume (t) | 249 | 158 |
| Total applied acetamiprid (kg) | 49 | 31.6 |
| acetamiprid per area (μg/$m^2$) | 70 | 45 |
| Number of patients | 78 | 63 |
| male/female | 20/58 | 18/45 |
| Age (years old) | 2-62 | 3-78 |
| under 15 years old | 32 (50%) | 15 (26%) |
| Abnormal Electrocardiogram (%) | 69 (89%) | 57 (91%) |
| Sinus tachycardia | 7 ( 9%) | 14 (22%) |
| Sinus bradycardia | 25 (32%) | 4 ( 6%) |
| ST-T changes | 53 (68%) | 30 (49%) |
| T-wave abnormality | 16 (21%) | 7 (11%) |
| Prolonged QT intervals | 7 ( 9%) | 12 (19%) |

**Table 3 Acetamiprid deposits during and after the ground application**

| Sampling point | Distance from the sprayed area (km) | Maximum Deposit ($ng/cm^2$) | Interval after application (hours) |
|---|---|---|---|
| Ⅰ | 0 | 311.7 | During application |
| Ⅱ | 0.5 | 11.2 | During application |
| Ⅲ | 2 | 1.31 | 6-8 |
| Ⅳ | 3 | 2.32 | During application |
| Ⅴ | 5 | 2.25 | 6-8 |

Reference: 15)

and dizziness[7]. Electrocardiographic abnormality was found in 91% patients (ST-T change, tachycardia, QT prolongation, bradycardia) (Table 2).

In 2005, a research group quantified acetamiprid deposits after the ground application. The maximum values of deposits in the applied area were higher than those in non-applied area; however, low-level deposits were also found at 2-5 km away from the applied area. The maximum values of deposits were sometimes observed 6-8 hours after the application (Table 3)[15]. If the patient's symptoms were caused by acetamiprid, the maximum dose of exposure is estimated to be 84.1μg/kg BW.

In literature, a few cases of occupational inhalational exposure to NN insecticides were reported. In acute inhalational exposure, imidacloprid caused severe systemic action, e.g. renal and hepatic dysfunctions, leukoclastic vasculitis, rhabdomyolysis, for more than a week[16, 17]; and in chronic inhalational exposure, NN was related to a decrease in lung capacity[18]. Inhalational exposure to NN may induce a delayed reaction, which is consistent with the animal studies[19, 20].

### ⅱ. Subacute intoxication after consecutive intake of fruits and tea beverages

Since August 2006, 1,111 patients visited the clinic in eight months with chest pain, chest discomfort or palpitations, which resembled to those found after acetemiprid spray application. They also complained of headache, abdominal pain, muscle pain and fever. Impaired short-term memory, finger tremor, and abnormal heart rhythm (tachycardia, bradycardia and arrhythmia) were observed clinically. The patients' ages ranged from 1 to 79 years old. All patients were non-smokers. It is noteworthy that acetemiprid spray application for pinewoods ceased by July 2006 in Gunma. Five hundred forty nine out of 1,111 of the patients had consumed large amounts of conventionally grown domestic fruits/ vegetables or tea beverages before the onset. All were recovered by prohibition of domestic fruits and tea beverage[7]. We therefore referred to these new symptoms as neo-Conventional Produce Syndrome (neo-CPS), which follows the intake of conventional produce. From 2007 to 2009, no less than 4,000 patients visited the clinic with neo-CPS, and 193 were from outside of Gunma, e.g. Saitama, Tochigi, Tokyo, Kanagawa, Ibaragi, Chiba, Nagano, Niigata, Yamanashi, Shizuoka, Aichi, Miyagi, Iwate, Osaka and Shimane.

We speculated neo-CPS might be mainly caused by oral NN exposure, although exposure to other pesticides, phytochemicals and other environmental pollutants has not been excluded. Neo-CPS was empirically defined as the symptoms of unknown origin after consecutive intake of domestic fruits/ vegetables 500g/day or tea beverage 500mℓ/day for more than several days. We began to collect neo-CPS patients' urine for chemical analysis of NN metabolites.

Firstly, we sought to analyze 6-chloronicotinic acid (6-CNA) as a urinary biomarker by ion chromatography with the lowest level of qualification (LOQ) of 40 ppb. 6-CNA is a common metabolite of chloropyridinyl

neonicotinoid i.e. acetamiprid, imidacloprid, thiacloprid, nitenpyram[21] and cycloxaprid (Figure 3). Urine was prospectively collected from thirty-three patients with neo-CPS. They were classified into two groups by the onset time, Group A: eleven patients who visited the clinic within 24 hours after the onset, Group B: twenty-two patients who visited the clinic one to several days after the onset or whose onset time was not identified. In Group B, urinary 6-CNA was detected from only one patient. In Group A, urinary 6-CNA was detected from six of eleven patients who showed similar symptoms and mainly took domestic fruits or tea beverage before the onset[21] (Figure 4). We thought those symptoms were one of the core group of neo-CPS which related to chloropyridinyl neonicotinoid exposure, and named them neo-nicotinic symptoms, i.e. finger tremor, impaired short-term memory, fever (>37℃), general fatigue, headache, palpitation/chest pain, abdominal pain, muscle pain/muscle weakness/muscle spasm, and cough.

Secondly, we quantified urinary 6-CNA from neo-CPS patients with neo-nicotinic symptoms by liquid chromatography/mass spectrometry with LOQ of 1 ppb. The levels of urinary 6-CNA ranged from 7.5 to 84.8 ppb. Interestingly in five of six cases, the maximum level of 6-CNA of each patient's urine was detected 2-20 days after the first visit[21].

Thirdly, we analyzed the known metabolites of acetemiprid, imidacloprid and clothianidin by liquid chromatography/time-of-flight mass spectrometry in the urine of three neo-CPS patients with neo-nicotinic symptoms within 24 hours from onset. NN and metabolites were qualitatively found from two of them as follows: acetemiprid; an acetamiprid metabolite, *N*-desmethyl-acetamiprid; three imidacloprid metabolites, 5-hydroxy-imidacloprid, 4,5-dehydro-imidacloprid and 4,5-dihydroxy-imidacloprid; two clothianidin metabolites, *N*-desmethyl-clothianidin, *N*-(2-(methylsulfinyl) thiazole-5-carboxyl)-glycine; and a common metabolite of acetemiprid and imidacloprid, *N*- (6-chloronicotinoyl)-glycine[4] (Figure 3).

Finally, we quantified *N*-desmethyl-acetamiprid at 3.2 ppb with LOQ of 0.5 ppb, and acetemiprid at 0.058 ppb (less than LOQ of 0.1 ppb) by liquid chromatography/tandem mass spectrometry in the urine of a neo-CPS patient with neo-nicotinic symptoms. *N*-desmethyl-acetamiprid was quantified no more than LOQ from eleven patients without neo-nicotinic symptoms[4]. From the urine of three 60s-70s women of neo-CPS without abnormal ECG, who were diagnosed dementia by low value of Hasegawa Dementia Scale (<20) with impaired short-term memory, 0.91-1.7 ppb of *N*-desmethyl-acetamiprid was quantified (unpublished data).

We speculate that neo-nicotinic symptoms is related to the accumulation of NN in the human body,

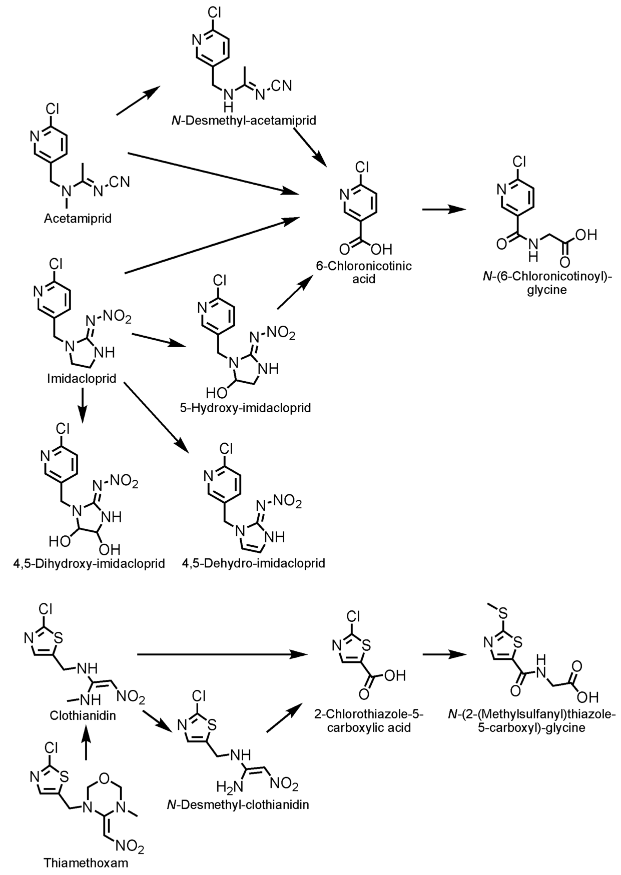


**Figure 3 Metabolic transformation of acetemiprid, imidacloprid, clothianidin and selected metabolites.**

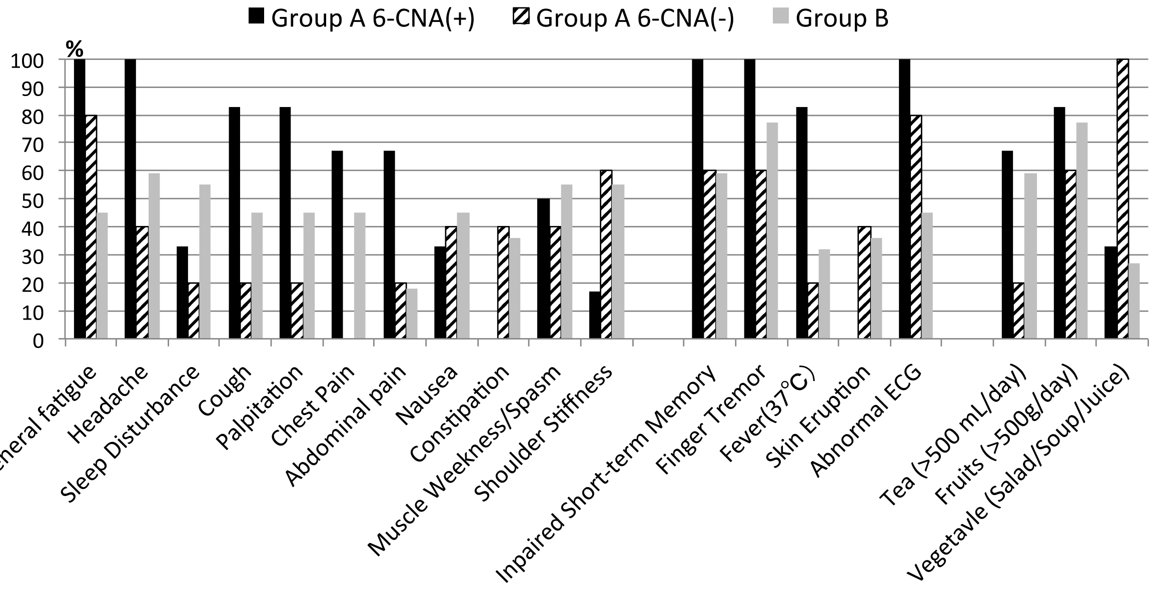


**Figure 4 Comparison of subjective symptoms, clinical findings and fruits/vegetables/ tea beverage intake between three groups of neo-CPS.**

especially acetamiprid. Although it remains unknown whether NNs, as well as metabolites, are causative toxicants or coincidental xenobiotics, we found retrospectively 102 patients (male/female=20/82) with neo-nicotinic symptoms from 2008 to 2012, all were neo-CPS, and recovered by stopping the intake of domestic fruits and tea beverage.

The reversibility of impaired short-term memory is one of the most representative symptoms of neo-CPS. In October 2006, a 14-year-old girl visited the clinic with headache, shoulder pain, palpitations, impaired short-term memory, insomnia and a poor result of examination. Her grades at school were getting worse. She descended from the top of her class to the bottom in a few months. She could not remember what she ate the previous day. In November 2006, she visited an ophthalmologist, and was diagnosed with accommodation disorder suspected by pesticide exposure. In January 2007, her headache continued. The doctor stopped her intake of fruits and tea beverages. One week later, her headache disappeared; and her impaired short-term memory and disorder of accommodation improved. Her performance improved gradually; and her grades were at the top of her class again (unpublished data).

Finger tremor is another typical symptom of neo-CPS. Neurologically, it is postural tremor of approximately 4-12 Hz, and may be diagnosed as an idiopathic one. As the diagnosis of essential tremor of non-smoker, not only caffeine but also the intake of conventional produces might be considered for exclusion.

Characteristic electrophysiological abnormalities are observed in neo-CPS patients. A 28-year-old woman visited the clinic with chest pain, headache, sleepiness, constipation, throat pain, and muscle pain after consecutive intake of bottled green tea 500 mℓ per day for more than 3 months. Finger tremor and impaired short-term memory were also observed. In her ECG, severe sinus tachycardia (146 bpm) was observed on the first day of visit. On the next day, her heart rate returned to almost the normal range, but mild QT prolongation emerged. Tachycardia followed by bradycardia is popular in the patients with neo-nicotinic symptoms, as well as intermittent WPW syndrome, paroxysmal atrial fibrillation or

early repolarization, which is a candidate trigger of sudden death. NN might facilitate those kinds of arrhythmia by the substantial electrocardiographic condition. In auditory brain stem response (ABR), the shortening of the interval between the first wave and the fifth wave (I-V interval) is a typical finding. By sound stimulation to the ear, five waves may occur by the successive nerve excitation. It is known that in chronic OP intoxication the fifth wave latency and I-V interval are elongated, but in neo-CPS with neo-nicotinic symptoms, both shorten. In the infrared pupillography, not only sympathotonic but also parasympathotonic findings were observed. A pupil action for photo-stimulation is a good marker of autonomic activity. Her heart rate was 146 bpm, which suggests sympathotonic. However, the parameter of infrared pupillography, D1 and A1 decreased, which means miosis; and it can be interpreted as parasympathetic dominant state. Input disturbance is unlikely. Maximum dilatation velocity increased[22].

Attention deficit hyperactivity disorder (ADHD)-like reactions were observed in a few children of neo-CPS. A 3-year-old boy went for grape picking with his parents. From Sep 4$^{th}$, he started drinking fresh grape juice more than 500 mℓ per day. On Sep 8$^{th}$, he showed incontinence, stomachache and low activity; and his heart rate was 108 bpm. On Sep 11$^{th}$, he was hyperactive and unruly; and his heart rate was 85 bpm. On Sep 16$^{th}$, he recovered completely, and his ADHD-like action diminished (unpublished data). We could not analyze his urine.

Skin eruption was frequently observed in neo-CPS who classified in Group B[20]. Anti-virus drug were effective for the skin eruption. In Japan, the pandemic of Kaposi's varicellimorm eruption, which is caused by Herpes Simplex virus, was reported[23]. The immunotoxicity of NNs would be considered for human being after chronic exposure. Di Prisco reported that clothianidin acts as a negative modulator of NF-κB activation in honey bees[24].

Depression was also observed in a patient of neo-CPS. A 31-year-old man drank bottled green tea 500mℓ per day consecutively. Since he had prolonged exposure to imidacloprid by the termite control of his dwelling in 2003, he was depressive. A psychiatric doctor administered antipsychotic drugs in diagnosis of depression with personality disorder. One day, he visited the clinic and complained of muscle pain, finger tremor, general fatigue, impaired short-term memory, insomnia, poor verbal communication, palpitations, chest pain, and fever 38.5 °C. His ECG showed early repolarization pattern. In auditory brainstem response, I-V interval was shortened, and pupillography showed sympathotonic and parasympathotonic pattern. After six weeks of the prohibition of green tea, his verbal communication improved; and the irritation diminished[22].

### iii. Toxicological consideration of neo-Conventional Produce Syndrome

Minimum levels of urinary NNs and metabolites in neo-CPS are as follows: *N*-desmethyl-acetamiprid: 3.2 ppb in neo-nicotinic symptoms and 0.91 ppb in dementia; acetamiprid: 0.058 ppb (<LOQ) in neo-nicotinic symptoms. In the literature, the minimum toxic dose of acute acetemiprid intoxication was rather high (30 mg/kg BW) with plasma acetemiprid of 2.39 ppm[7]. However, 3 ppb of imidacloprid was detected from the urine of a patient who attempted suicide by ingestion of imidacloprid solution and was put in intensive care[25].

neo-nicotinic symptoms seems to occur by the mediation of nicotinic receptors, because heart, central nervous systems, neuromuscular junction, gastrointestinal tract, keratinocyte and immune system have nicotinic acetylcholine receptors. The symptoms developed systemically after the onset and diminished slowly by prohibition of conventional produce. Bioaccumulation of NN metabolite may occur in humans by consecutive intake of NNs contaminated food, because bioaccumulation of imidacloprid by low dose exposure was observed in animal study[26].

**Table 4 MRLs for apples and tea-leaves**

| Apples | Japan | US | CODEX | EU |
|---|---|---|---|---|
| Imidacloprid | 0.5 | 0.5 | 0.5 | 0.5 |
| Acetamiprid | 2 | 1 | | 0.7 |
| Thiacloprid | 2 | 0.3 | 0.7 | 0.3 |
| Thiamethoxam | 0.3 | 0.2 | 0.3 | 0.3 |
| Clothianidin | 1 | 0.5 | 0.5 | 0.5 |
| Nitenpyram | 0.5 | | | 0.01* |
| Dinotefuran | 0.5 | | | 0.01* |

| Tea-leaves | Japan | US | CODEX | EU |
|---|---|---|---|---|
| Imidacloprid | 10 | – | – | 0.05* |
| Acetamiprid | 30 | 50** | – | 0.1* |
| Thiacloprid | 30 | – | – | 10 |
| Thiamethoxam | 20 | 20*** | 20 | 20 |
| Clothianidin | 50 | 70**** | 0.7 | 0.7 |
| Nitenpyram | 10 | – | – | – |
| Dinotefuran | 25 | – | – | – |

* indicates lower limit of analytical determination.
**There are no U.S. registrations as of February 10, 2010, for the use of acetamiprid on dried tea-leaves.
***There are no U.S. registrations as of March 27, 2013.
****No U.S. registrations.

In our study, women seem to be more susceptible than men[4, 7, 21]. Because both cytochrome P-450 enzymes and aldehyde oxidases are active for NN metabolism in the human body, they may develop some metabolites whose toxicities are not comprehensively evaluated; and the diversity of those enzyme activities by species, polymorphism and estrogen-like substances are known[27–29].

As the cause of neo-CPS, other pesticides, phytochemicals and new environmental pollutants must be considered, in addition to nine neonicotinoids. Further investigation is needed.

## Ⅳ. Why neonicotinoid intoxication occurs in Japan?

### ⅰ. Poor understanding of characteristics

An advertising copy of NN insecticides is "safe and mild". In fact, deaths by acute neonicotinoid poisoning are rare in Japan because concentrations of NNs in liquid formulations are low[7]. However, acute oral toxicities of NNs are comparable to those of OPs, and lethal (Table 1). We have not been aware of the high residual pesticides for the decades before 1992 when the use of NNs started. Successive use of NNs may cause accumulation and toxicity in the environment and human beings. We should use NN insecticides restrainedly and rationally.

### ⅱ. Vicinity of applied area from residence

Many farms and forests in Japan are located near residential areas. When NN insecticide is applied by aerial or ground spraying, residents are vulnerable to exposure to drift. The information of spray drift caused by NN application should be properly obtained and needs to be disclosed to the public.

### ⅲ. High MRLs and a broad range of application

The maximum residue levels (MRL) for acetamiprid are very high for fruits and tea-leaves in Japan. Comparisons of MRLs of several NNs for some food are shown in Table 4. The MRLs of imidacloprid and thiamethoxam are almost at the same level in the

world; however the MRLs of acetamiprid, thiacloprid, and clothianidin are higher in Japan than the EU and the US[30–32]. The use of NN insecticides should be fully controlled and recorded; and NN exposure in the general population should be monitored on a systematic basis with appropriate biomarkers, such as 6-CNA.

## Ⅴ. Conclusion

In Japan, environmental exposure to neonicotinoid insecticides is prevalent. We have a rather high risk for environmental intoxication due to the poor understanding of its characteristics, the vicinity of the applied areas from residence, the high MRLs and the broad range of application. Neonicotinoids are not necessarily safer than organophosphates. The use of neonicotinoid insecticides should be modest and needs to be regulated according to the precautionary principle with full respect for human health.

### Acknowledgements

I wish to thank to all of my co-workers, Yoshiko Aoyama at Aoyama Allergy Clinic, Kazutoshi Fujioka at Hawaii Institute of Molecular Education, Motoyuki Kamata at Kanto-Gakuin University, Toru Aoi at Gunma National College of Technology, Tomonori Kawakami at Toyama Prefectural University, Tomiko Yoshihara at Tokyo Kasei University, Naomichi Moribayashi at Kiryu Kosei General Hospital, Mayumi Ishizuka, Yoshinori Ikenaka, and Shyota Nakayama at Hokkaido University, Kensaku Imai and Yoshifumi Ujiie at Genaris, Inc. I thank to Professor Makiko Komori at Tokyo Women's Medical University for completing the ethical procedure to collect human urine samples.

### Funding

This study is partially supported by Japan Endocrine-disruptor Preventive Action. The funders had no role in study design, data collection and analysis, decision to publish, or preparation of the manuscript. No additional external funding received for this study.

### Conflict of interest

The author has declared that no competing interest exists.

## References

1 ) http://eur-lex.europa.eu/LexUriServ/LexUriServ.do?uri=OJ:L:2013:139:0012:0026:EN:PDF (2013.12.1)

2 ) http://kokumin-kaigi.sakura.ne.jp/kokumin/wp-content/uploads/Neonicotinoid_e.pdf (2013.12.1)

3 ) Taira K, Aoyama Y. Electrocardiographic changes in patients with cardiac symptoms and headache in the area where neonicotinoid and organophosphate insecticides were spread in 2005. Jpn J Clin Ecol 15:114-123, 2006 (*in Japanese*)

4 ) Taira K, Fujioka K, et al. Qualitative Profiling and Quantification of Neonicotinoid Metabolites in Human Urine by Liquid Chromatography Coupled with Mass Spectrometry. PLoS One. 2013 Nov 12; 8(11): e80332

5 ) Shao X, Swenson TL, et al. Cycloxaprid insecticide: nicotinic acetylcholine receptor binding site and metabolism. J Agric Food Chem 2013 Aug 7; 61:7883-7888

6 ) Watson GB, Loso MR, et al. Novel nicotinic action of the sulfoximine insecticide sulfoxaflor. Insect Biochem Mol Biol 41:432-439, 2011

7 ) Taira K. Health effects of neonicotinoid insecticides -Part 1: Physicochemical Characteristics and Case Reports- Jpn J Clin Ecol 21: 24-34, 2012 (*in Japanese*)

8 ) Japan Plant Protection Association: Handbook of Pesticide, Japan Plant Protection Association. 2011, pp 65-75 (*in Japanese*)

9 ) Food Safety Commission of Japan, Ed. Noyaku Hyokasyo (Pesticide Evaluation Report) Acetamiprid. Food Safety Commission of Japan, Tokyo. 2010 Aug 12 (*in Japanese*)

10) http://toxnet.nlm.nih.gov/cgi-bin/sis/search/a?dbs+hsdb:@term+@DOCNO+200 (2014.2.6)

11) http://toxnet.nlm.nih.gov/cgi-bin/sis/search/a?dbs+hsdb:@term+@DOCNO+1590 (2014.2.6)

12) http://toxnet.nlm.nih.gov/cgi-bin/sis/search/a?dbs+hsdb:@term+@DOCNO+7274 (2013.12.1)

13) Coscollàa C, Yusàa V, et al. Multi-residue analysis of 30 currently used pesticides in fine airborne particulate matter (PM 2.5) by microwave-assisted extraction and liquid chromatography-tandem mass spectrometry. J Chromatogr A. 1216: 8817-8827, 2009

14) Yamamoto A, Terao T, et al. Evaluation of river pollution of neonicotinoids in Osaka City (Japan) by LC/MS with dopant-assisted photoionization. J Environ Monit. 14: 2189-2194, 2012

15) Ichikawa Y, Moriyama M, et al. Drift monitoring of acetamiprid sprayed by an air-blaster to pine trees in Gunma Pref. Nihon Nohyaku Gakkaishi 33:281–288, 2008 (*in Japanese*)

16) Agarwal R, Srinivas R. Severe neuropsychiatric manifestations and rhabdomyolysis in a patient with imidacloprid poisoning. Am J Emerg Med. 25: 844-845, 2007
17) Agha A, Bella A, et al. Imidacloprid poisoning presenting as leukoclastic vasculitis with renal and hepatic dysfunction. Saudi J Kidney Dis Transpl 23: 1300-1303, 2012
18) Hernandez AF, Casado I, et al. Low level of exposure to pesticides leads to lung dysfunction in occupationally exposed subjects. Inhal Toxicol 20: 839-849, 2008
19) Nihon-Soda: Noyaku-Shoroku Acetamiprid, Ⅷ.Toxicity A5-8, http://www.acis.famic.go.jp/syouroku/acetamiprid/acetamiprid_05.pdf (2014.1.4)
20) Sumitomo-Chemical: Noyaku-Shoroku Clothianidin, 2007, pp122-3, http://www.acis.famic.go.jp/syouroku/clothianidin/clothianidin_02.pdf (2014.1.4)
21) Taira K, Aoyama Y, et al. Detection of chloropyridinyl neonicotinoid insecticide metabolite 6-chloro-nicotinic acid in the urine: Six cases with subacute nicotinic symptoms. Chudoku Kenkyu 24: 222-230, 2011 (*in Japanese*)
22) Taira K, Moribayashi N, et al. Nicotinic cholinergic symptoms after consecutive tea drink consumption: Clinical findings of electrocardiography, auditory brainstem response, and infrared pupillography, and acetamiprid residual analysis. Jpn J Clin Ecol 18:19-34, 2009
23) Kimata H. Rapidly increasing incidence of Kaposi's varicelliform eruption in patients with atopic dermatitis. Indian J Dermatol Venereol Leprol 74: 260-261, 2008
24) Di Prisco G, Cavaliere V, et al. Neonicotinoid clothianidin adversely affects insect immunity and promotes replication of a viral pathogen in honey bees. Proc Natl Acad Sci U S A. 2013 Oct 21. [Epub ahead of print]
25) Tamura M, Endo Y, et al. Investigation and case study of Imidacloprid insecticide caused poisoning. Chudoku Kenkyu 15: 309-312, 2002 (*in Japanese*)
26) Kavvalakis MP, Tzatzarakis MN, et al. Development and application of LC-APCI-MS method for biomonitoring of animal and human exposure to imidacloprid. Chemosphere 93: 2612-2620, 2013
27) Shi X, Dick RA, et al. Enzymes and inhibitors in neonicotinoid insecticide metabolism. J Agric Food Chem 57: 4861-4866, 2009
28) Schofield PC, Robertson IG, et al. Inter-species variation in the metabolism and inhibition of N-[(2'-dimethylamino)ethyl] acridine-4-carboxamide (DACA) by aldehyde oxidase. Biochem Pharmacol 59: 161-165, 2000
29) Obach RS, Huynh P, et al. Human liver aldehyde oxidase: inhibition by 239 drugs. J Clin Pharmacol 44: 7-19, 2004
30) http://db-out.nies.go.jp/kis-plus/index_3.html (2013.12.1)
31) http://ec.europa.eu/sanco_pesticides/public/?event=homepage&CFID=894021&CFTOKEN=42287421&jsessionid=0904271dd66640a3f2a4706d1442172c6e74TR (2013.12.1)
32) http://www.ecfr.gov/cgi-bin/text-idx?c=ecfr&tpl=%2Findex.tpl (2013.12.1)

# 日本人のネオニコチノイド曝露

## 要約

ネオコチノイド系殺虫剤は、日本では92年から使われ、09農薬年度の出荷量は407tだった。水溶性、浸透性、低揮発性、熱安定性で、作物、土壌、自然水中で持続的に効力を発揮し、摂取後、腸管粘膜、脳血液関門、胎盤を通過する。使用増加に伴い、環境中毒と思われる症例が見出されている。

04年、05年にアセタミプリドの地上散布後、各78、63人が受診し、頭痛、筋痛、胸痛、発熱、手指振戦、記憶障害、心電図異常がみられた。

06年以降、国産果物・野菜500g/日または茶飲料500mℓ/日以上を連日摂取し体調不良を訴えた患者が4,000人以上受診し、うち可逆性の短期記憶障害、手指振戦、心電図異常、頭痛、全身倦怠、発熱、咳、動悸、胸痛、腹痛、筋痛/筋攣縮を訴えた患者の尿中からネオニコチノイド代謝産物の6-クロロニコチン酸7.5-84.8ng/mℓ、N-デスメチル-アセタミプリド3.2ng/mℓを検出した。 (臨床環境 23：14－24, 2014)

《キーワード》ネオニコチノイド、殺虫剤、アセタミプリド、イミダクロプリド、中毒
